# Spaceflight effects on human vascular smooth muscle cell phenotype and function

**DOI:** 10.1038/s41526-024-00380-w

**Published:** 2024-03-28

**Authors:** Marina M. Scotti, Brandon K. Wilson, Jodi L. Bubenik, Fahong Yu, Maurice S. Swanson, Josephine B. Allen

**Affiliations:** 1 Department of Materials Science and Engineering, University of Florida, Gainesville, FL USA; 2 Department of Biomedical Engineering, University of Florida, Gainesville, FL USA; 3 Department of Molecular Genetics and Microbiology, Center for NeuroGenetics, University of Florida, Gainesville, FL USA; 4 Interdisciplinary Center for Biotechnology Research, University of Florida, Gainesville, FL USA

**Keywords:** Molecular biology, Cell biology

## Abstract

The cardiovascular system is strongly impacted by the hazards of spaceflight. Astronauts spending steadily increasing lengths of time in microgravity are subject to cardiovascular deconditioning resulting in loss of vascular tone, reduced total blood volume, and diminished cardiac output. Appreciating the mechanisms by which the cells of the vasculature are altered during spaceflight will be integral to understanding and combating these deleterious effects as the human presence in space advances. In this study, we performed RNA-Seq analysis coupled with review by QIAGEN Ingenuity Pathway Analysis software on human aortic smooth muscle cells (HASMCs) cultured for 3 days in microgravity and aboard the International Space Station to assess the transcriptomic changes that occur during spaceflight. The results of our RNA-Seq analysis show that SMCs undergo a wide range of transcriptional alteration while in space, significantly affecting 4422 genes. SMCs largely down-regulate markers of the contractile, synthetic, and osteogenic phenotypes including smooth muscle alpha actin (αSMA), matrix metalloproteinases (MMPs), and bone morphogenic proteins (BMPs). Additionally, components of several cellular signaling pathways were strongly impacted including the STAT3, NFκB, PI3K/AKT, HIF1α, and Endothelin pathways. This study highlights the significant changes in transcriptional behavior SMCs exhibit during spaceflight and puts these changes in context to better understand vascular function in space.

## Introduction

Space flight exposes biological organisms to one of the most foreign and hostile environments accessible to human exploration. Several features, including microgravity, increased radiation exposure, alterations in circadian rhythms, etc. have been shown to produce both individual and synergistic effects across physiology^1^. The numerous physiological changes that occur during space flight require characterization, particularly at the cellular level. Many cell types have been cultured in both real and simulated microgravity including various stem cells, macrophages, osteoblasts, and retinal cells to better understand the isolated changes that these cells undergo while in space^2–6^. However, the smooth muscle cell population of the vasculature remains understudied.

Microgravity exposure is well known to cause muscular atrophy, bone resorption, flattening of the eye, and more^7^. Some of the more significant maladaptations to arise from space flight are found in the cardiovascular system. Initially, blood disseminates to a more uniform distribution throughout the body, expanding the vessels of the upper torso and head to accommodate the presence of increased blood volume. Additionally, the smooth muscle that lines the vascular system and composes the heart loses tone. Overall, this state is referred to as cardiovascular deconditioning and can have serious implications for astronaut health^8–11^. While these large-scale features of cardiovascular deconditioning are known, behavioral changes at the cellular level require more investigation, i.e., changes in cytokine production, cellular phenotype transition, and angiogenetic or inflammatory potential. Understanding these fundamental changes in cellular homeostasis will be critical to the safe and mindful pursuit of space travel.

Vascular smooth muscle cells (VSMCs) play a critical role in the maintenance of vascular homeostasis, controlling vascular tone, vascular remodeling, and inflammation. To accomplish this VSMCs maintain a degree of phenotypic plasticity, allowing these cells to modulate their mechanical and chemical behavior to suit the fluctuating vascular environment. These phenotypes include contractile, synthetic, and osteogenic, among several others. VSMCs displaying a contractile phenotype conform to a spindle shaped morphology and produce components of the contractile apparatus including smooth muscle α-actin (*ACTA2*) and myosin heavy chain (*MYH11*), along with several accessory proteins like tropomyosin (*TPM1-4*). Synthetic VSMCs, in contrast, take on a rhomboid or cobblestone appearance while increasing the production of migratory proteins such as matrix metalloproteinases (MMPs), extracellular matrix (ECM) components, downregulation of the contractile apparatus, and increased proliferative capacity^12^. This propensity for vascular remodeling has implicated the synthetic VSMC phenotype in the progression of several vascular diseases including atherosclerosis^13^. The impact of spaceflight on the phenotypic polarization of VSMCs has yet to be explored.

VSMC phenotypic switching is also characterized by altered ECM production and composition. Collagen type IV (Col IV), the primary component of the vascular basement membrane, has been shown to promote the contractile phenotype in rat smooth muscle cells^14^. Laminin also maintains the contractile phenotype with evidence supporting its involvement in the transition from synthetic to contractile phenotype^15,16^. In contrast, the heightened presence of collagen type I (Col I) and fibronectin promotes a synthetic phenotypic switch^17,18^. Additionally, synthetic VSMCs have been shown to produce larger amounts of ECM proteins compared to their contractile counterparts^12,19^.

Osteogenic VSMCs contribute to the vessel calcification and stiffening that occurs with age and with the progression of atherosclerosis. Similar to the synthetic phenotype, osteogenic phenotypic switching is characterized by a decrease in expression of the contractile apparatus. Additionally, several proteins normally expressed by osteoblasts are up-regulated including osteocalcin, osteonectin, bone morphogenic proteins (BMPs), ECM proteins like Col I, and the RUNX2 transcription factor^20–22^.

In this study, we probed the transcriptome of vascular smooth muscle cells cultured in microgravity and aboard the International Space Station (ISS). The results of this transcriptomic analysis were used to observe potential pathway regulation mechanisms via QIAGEN Ingenuity Pathway Analysis (IPA) software. Several critical pathways showed significant changes in gene expression related to proliferation and survival, migration, apoptosis, inflammation, and phenotypic switching. Here we present a comparison of the transcriptomes of vascular smooth muscle cells cultured in both real space microgravity and in standard normal gravity on earth, while highlighting the impact on select cellular signaling pathways.

## Results

### Post-flight sample assessments

Once returned to earth, the hardware units were visually inspected, and it was confirmed that each cassette functioned properly and delivered the loaded RNAlater to each cell population. The cryopreserved flight and ground control samples were thawed and treated to remove contaminating DNA which resulted in a yield of total RNA of 308.6 ± 154 ng and 121.5 ± 22.9 ng, respectively. This yield was sufficient to proceed with RNAseq using 100 ng RNA per sample. RNA quality was determined to be appropriate for subsequent RNAseq analysis as all samples passed the fragment analyzer test with an RNA integrity number (RIN) ≥ 7.

### Differential gene expression

**Fig. 1:**
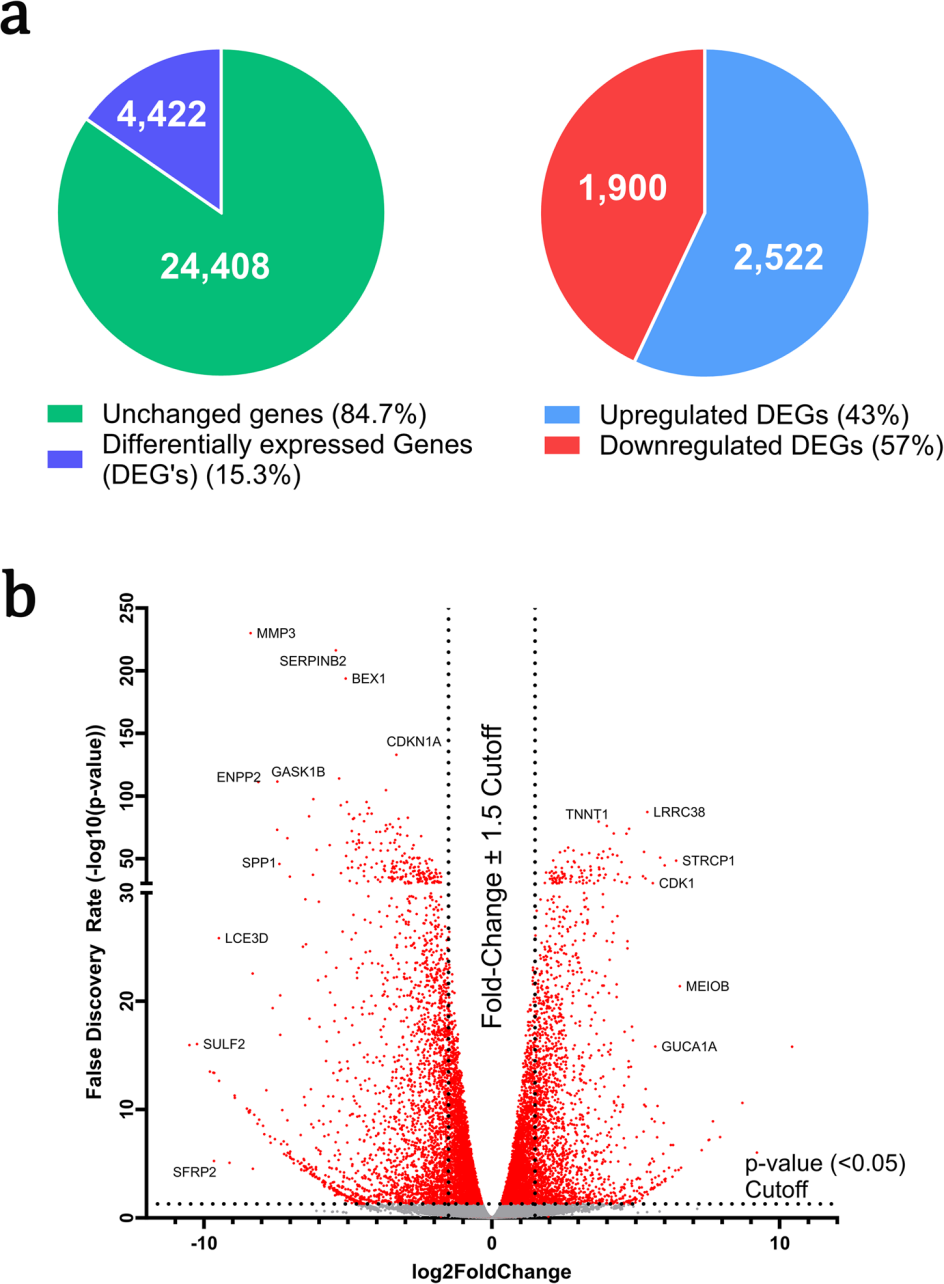
Summary overview of the results from RNAseq analysis. Analysis of all genes assessed (28,830). **a** Of the 28,830 genes assessed, 4422 were considered differentially expressed (adjusted *p*-value of ≤0.05, −1.5 ≥log2-fold change ≥1.5). Of those, 1900 were up-regulated (log2-fold change ≥1.5) and 2522 were down-regulated (log2-fold change ≤ −1.5). **b** Volcano plot of all 28,830 genes. Red indicates targets above the False Discovery Rate threshold of -log(p-value) = 1.3 (*p* ≤ 0.05). Vertical dotted lines indicate a log2-fold change cutoff of ±1.5.

A full transcriptomic analysis of the ground control and space flight VSMCs resulted in the differential expression of over 4,422 genes that surpassed the threshold of ±1.50 log2-fold change. Of these, 43% were up-regulated (log2-fold change ≥1.50) and 57% were down-regulated (log2-fold change ≤ −1.50) (Fig. 1a, b). Transcriptomic data was analyzed using the IPA software from QIAGEN to provide context for the expression of specific genes in terms of cellular signaling pathways. The IPA software compared our data to the QIAGEN Knowledge Base composed of over 100,000 public datasets while applying these comparisons to several canonical pathways, allowing us to make informed hypotheses in a biological context. The software also provided predictions on the regulation of downstream molecules and products based on the transcriptional changes seen in our cells.

**Fig. 2:**
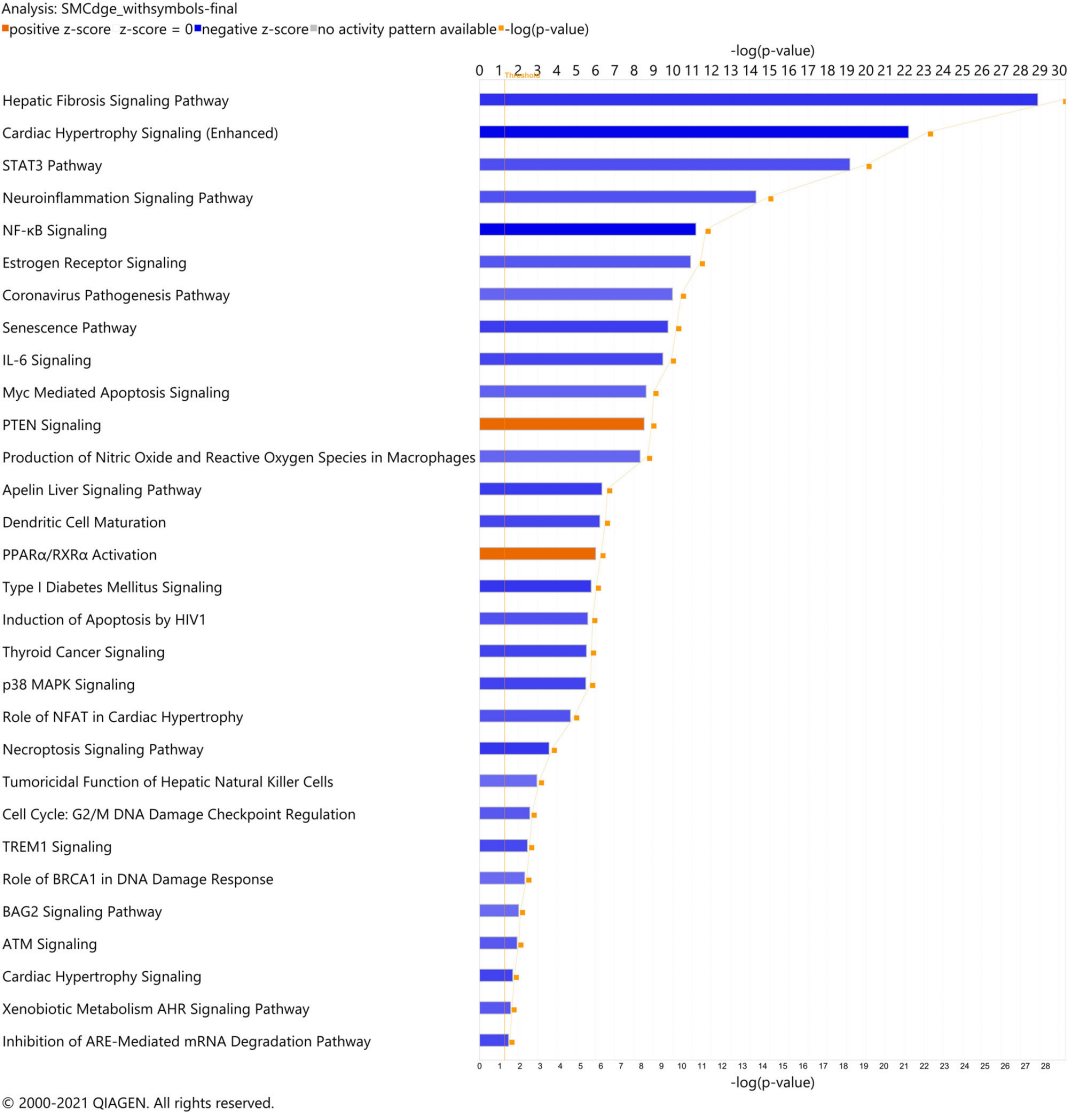
Canonical pathways predicted by Ingenuity Pathway Analysis. The canonical pathways are identified with a right-tailed Fisher’s Exact Test for *p*-value ≤ 0.05 and a z-score of ±2. The z-score is the predicted activation state of the canonical pathway. Blue or lighter blue color indicated a negative z-score and downregulation (inhibition), orange or light orange color indicate a positive z-score and upregulation (activation) of the pathway. The light orange line is the threshold for significance.

Canonical pathways were significantly enriched in the human smooth muscle cells. A total of 317 significant pathways (adjusted *p*-value < 0.05) were identified from the RNA-seq results. Considering z-score of ±2 for significant activation and inhibition status in the pathway analysis, only 30 of the total 317 pathways had absolute z-score more than 2.0, with 28 pathways as significantly inhibited and two pathways, PTEN signaling and PPARα/RXRα activation, as significantly activated. (Fig. 2 Differentially expressed genes (DEGs) involved in the pathways with 0 < z-score < 0 are summarized in Supplementary Table 1.

Significant networks associated with the differentially expressed genes in the human smooth muscle cells were identified by IPA. The 10 most significant gene networks with scores from 24 to 41 were identified in our comparisons (Supplementary Table 2), including three networks corresponding to cardiovascular disease and cardiovascular system development and function. In the network of [cardiovascular system development and function, cellular movement, organismal development] (Supplementary Fig. 1A), 13 genes were down-regulated and 6 genes up-regulated. In the network of [cardiovascular disease, cardiovascular system development and function, organismal development] (Supplementary Fig. 1B), 13 genes were down-regulated and 5 genes up-regulated. In the network of [cardiovascular system development and function, cell-to-cell signaling and interaction, hematological system development and function] (Supplementary Fig. 1C), 11 genes were down-regulated and 7 genes up-regulated.

Of the selected pathways, the STAT3 pathway (z-score: −2.357, -log(*p*-value) = 19.2), most of genes from our datasets were predicted as up-regulated, and only one gene, CISH, as down-regulated. All genes (25 genes) were predicted as up-regulated in the pathway of NF-κB signaling (z-score = −3.411, -log(p-value) = 11.2. In the pathway of PI3K/AKT signaling (z-score: −1.134, -log(p-value) = 6.49), all genes, except the gene CDKN1A, were predicted as up-regulated. In the HIF1α signaling (z-score: −1.3, −log(*p*-value) = 16.2), 27 genes were expected to be up-regulated and only 2 genes, HSPA5 and TP53, as down-regulated. In the pathway of endothelin-1 signaling (z-score: 0.258, -log(*p*-value) = 5.38), except the gene PTGER2 as down-regulated, all other genes were expected as up-regulated.

The IPA analysis also identified 22 enriched cardiovascular signaling pathways (*p* < 0.05). However, only 3 pathways were identified as significantly inhibited (z-score < −2.0) with none displaying pathway activation (z-score > 2.0). These inhibited cardiovascular signaling pathways include cardiac hypertrophy signaling, Role of NFAT in Cardiac Hypertrophy, and Cardiac Hypertrophy Signaling (Enhanced). Related DEGs for each pathway and their statistical information were shown in Supplementary Table 3.

### Gene ontology

**Fig. 3:**
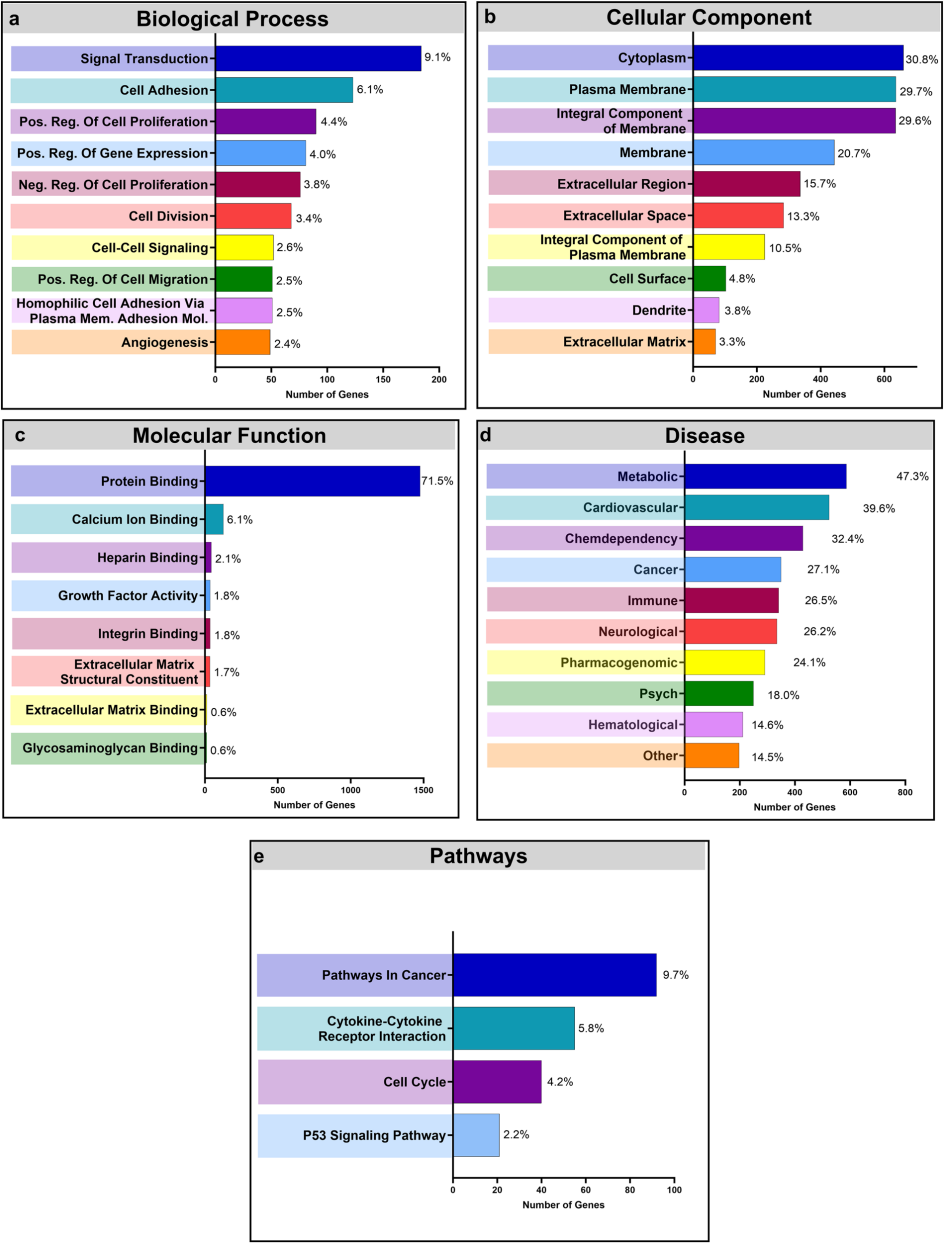
Gene ontology analysis of differentially regulated genes. DAVID analysis of DEGs of p-adj ≤ 0.05, −1.5 ≥ log2-fold change ≥ 1.5, and base mean expression ≥ 20. 2679 genes were uploaded with 2616 recognized by DAVID. Percents indicate the ratio of genes related to the specific annotation to the total number of assigned genes for the analysis. The ten highest GO terms representing **a** the Biological Process annotation; **b** the Cellular Component annotation; and **c** the Molecular Function annotation. **d** Ten highest represented disease annotations returned from the GAD Disease Classification analysis. **e** Significant pathways returned from KEGG analysis. Terms with a False Discovery Rate (FDR) ≤ 0.05 were treated as statistically significant.

GO, KEGG, and GAD analysis of significantly affected DEGs (adjusted *p*-value ≤ 0.05, −1.50 ≤ log2-fold change ≤ 1.50) with a base mean expression ≥20 was performed with the DAVID Bioinformatics Resource (Fig. 3). The GO annotations were supported by a comparative analysis with the PANTHER knowledgebase and were split into the 3 parent terms of biological process, cellular component, and molecular function. Biological process was assigned 2,025 DEGs with the top 10 most represented GO terms shown in Fig. 3a. Notable significantly affected terms include signal transduction (GO:0007165), cell adhesion (GO:0007155), and angiogenesis (GO:0001525). Cellular component (Fig. 3b) was assigned 2143 genes with the GO terms cytoplasm (GO:0005737), extracellular region (GO:0005576), extracellular space (GO:0005615), and extracellular matrix (GO:0031012) having a significant number of DEGs. Of the 2067 genes assigned to the molecular function category, the largest percentage of DEGs were related to the protein binding term (GO:0005515) with 1477 associated DEGs. Several other binding related terms were represented including calcium ion binding (GO:0005509), heparin binding (GO:0008201), integrin binding (GO:0005178), and extracellular matrix binding (GO:0050840) (Fig. 3c). Several GO terms discussed were also present in the PANTHER analysis including angiogenesis, extracellular matrix, and extracellular matrix binding. The comparative study is found in Supplementary Table 4. The GAD disease analysis (Fig. 3d) was assigned 1597 genes with 47.3% associated with the metabolic annotation. Other significant terms include cardiovascular, cancer, immune, and hematological. The KEGG pathway analysis (Fig. 3e) was assigned 949 genes with significant DEG representation under pathways in cancer, cytokine-cytokine receptor interaction, and cell cycle, and P53 signaling.

An additional analysis was performed using the DAVID and PANTHER databases separating the populations of upregulated or downregulated genes. This was done to gain a better understanding of which GO terms were associated with solely up or downregulation. Of the upregulated genes, the GO terms with the most associated genes were related to processes involving cellular division (GO:0051301, GO:0000278, GO:0007059, GO:0006260) (Supplementary Fig. 2), cellular components employed in mitosis (GO:0005813, GO:0000776, GO:0030496, GO:0072686) (Supplementary Fig. 2), and ATP metabolism (GO:0005524, GO:0016887) (Supplementary Fig. 2). The downregulated genes were primarily linked to the GO terms of signal transduction and cellular adhesion (GO:0007165, GO:0007155) (Supplementary Fig. 3), cellular membrane functions and interaction with external stimuli (GO:0005886, GO:0016020, GO:0005576) (Supplementary Fig. 3), and several binding processes including general protein and receptor binding (GO:0005515, GO:0005102). The upregulated genes returned no significant GAD classifications and the downregulated DEGs resembled the overall gene analysis, with the metabolic, cardiovascular, immune, cancer, and hematological terms being notably significant. Similarly, the downregulated KEGG pathway analysis was comparable to the overall gene analysis. The only KEGG pathway associated with the upregulated DEG population was the cell cycle term.

### VSMC phenotypic switching

Under normal conditions VSMCs maintain a quiescent contractile phenotype but may switch their phenotype in response to vascular damage or disease. During spaceflight, the transcription of numerous genes related to VSMC contractile, synthetic, and osteogenic phenotypes were altered. Contractile VSMCs regulate vascular tone through the contractile apparatus, a protein complex that allows for cellular contraction. Several components were downregulated (Table 1) including *ACTA2* (log2-fold change = −1.16), caldesmon 1 (*CALD1*) (log 2 fold change = −1.23), talin 2 (*TLN2*) (log2-fold change = −1.25), and filamin A (*FLNA*) (log2-fold change = −1.44), while calponin 1 (*CNN1*) (log2-fold change = 1.18) while α-actinin 3 (*ACTN3*) (log2-fold change = 3.45) was up-regulated. The general trend towards downregulation of the contractile apparatus in VSMCs suggests a potential loss in vascular contractile ability during spaceflight.

**Table 1:** Genes associated with VSMC phenotype

Function	Gene symbol	Annotation	Log2 fold change	padj
Contractile apparatus	ARPC4	Actin Related Protein 2/3 Complex Subunit 4	−1.46	8.47E−20
	FLNA	Filamin A	−1.44	1.55E−12
	TLN2	Talin 2	−1.25	3.12E−05
	ACTA2	Smooth Muscle a-Actin	−1.16	1.98E−09
	SRC	Proto-Oncogene c-Src	1.05	1.68E−12
	CNN1	Calponin 1	1.18	0.0003
	VIM	Vimentin	1.55	1.49E−19
	ACTN3	Actinin Alpha 3	3.45	0.0001
Extracellular matrix	LUM	Lumican	−6.48	3.94E−30
	DCN	Decorin	−4.70	1.77E−62
	COL3A1	Collagen Type III Alpha 1 Chain	−2.21	0.0028
	LAMA1	Laminin Subunit Alpha 1	−1.68	1.96E−21
	COL5A3	Collagen Type V Alpha 3 Chain	−1.64	4.24E−06
	VCAN	Versican	−1.46	1.44E−11
	ELN	Elastin	−1.27	0.0001
	NID2	Nidogen 2	−1.08	0.0009
Matrix metalloproteinase	MMP3	Matrix Metalloproteinase 3	−8.38	1.14E−230
	MMP15	Matrix Metalloproteinase 15	−5.06	8.31E−53
	MMP24	Matrix Metalloproteinase 24	−2.88	3.83E−07
	MMP28	Matrix Metalloproteinase 28	−2.65	0.0004
	MMP9	Matrix Metalloproteinase 9	−2.57	5.15E−08
	MMP14	Matrix Metalloproteinase 14	−1.41	8.26E−13
	MMP2	Matrix Metalloproteinase 2	−1.22	6.95E−09
	MMP10	Matrix Metalloproteinase 10	−1.20	0.0159
	MMP25	Matrix Metalloproteinase 25	1.93	0.0104
Osteogenic	SPP1	Osteopontin	−7.38	1.37E−46
	BMP4	Bone Morphogenic Protein 4	−3.58	3.85E−09
	COL2A1	Collagen Type II Alpha 1 Chain	−3.37	0.0016
	TNFRSF11B	Osteoprogerin	−2.95	1.10E−06
	BMP2	Bone Morphogenic Protein 2	−2.58	2.09E−51
	ALPL	Alkaline Phosaphatase	−2.41	1.25E−06
	WNT2	Wnt Family Member 2	−2.22	0.0019
	MGP	Matrix Gla Protein	−2.06	2.38E−07
	BMP6	Bone Morphogenic Protein 6	−1.59	1.56E−06
	VCAN	Versican	−1.46	1.44E−11
	SPARC	Osteonectin	−1.17	2.15E−07
	ACTA2	Smooth Muscle a-Actin	−1.16	1.98E−09
	RUNX2	RUNX Family Transcription Factor 2	0.45	0.0237
	WNT3	Wnt Family Member 3	1.04	1.13E−05
	BGLAP	Osteocalcin	1.59	1.21E−07
	WNT16	Wnt Family Member 16	3.81	9.37E−07

Synthetic VSMCs are defined by their enhanced ECM production, migration, and proliferation^23,24^. The transcription of several basement membrane ECM components was altered during spaceflight with the remaining majority displaying little significant change in comparison to the ground control (Table 1). Two isoforms of collagen type IV, the primary collagen component of the vascular basement membrane (BM), were strongly up-regulated. *COL4A3* (log2-fold change = 3.99) and *COL4A4* (log2-fold change = 2.23). Laminin, the main proteoglycan component of the BM, experienced both down and upregulation of its isoforms, *LAMA1* (log2-fold change = −1.68), *LAMA3* (log2-fold change = 2.29), *LAMA5* (log2-fold change = 2.05). The remaining alpha, beta, and gamma subunits were not significantly changed. Two other proteoglycans known to be expressed in synthetic VSMCs^25^, lumican (*LUM*) (log2-fold change = −6.48) and decorin (*DCN*) (log2-fold change = −4.70), were strongly down-regulated. A majority of collagen types and proteoglycans remained transcriptionally unchanged during spaceflight. Synthetic VSMCs have also been shown to increase production of MMPs, enzymes that degrade the ECM during migration. Nearly all MMPs were down-regulated during space flight (Table 1), notably MMP3 (log2-fold change = −8.38), MMP15 (log2-fold change = −5.06), MMP24 (log2-fold change = −2.88), and MMP28 (log2-fold change = −2.65). MMP25 was the only MMP enzyme to be significantly up-regulated (log2-fold change = 1.93).

A majority of osteogenic VSMC markers were down-regulated with exposure to spaceflight (Table 1). These include BMP 2, 4, and 6 (log2-fold change = −2.58, −3.58, −1.59), Osteopontin (*SPP1*) (log2-fold change = −7.38), Osteoprogerin (*TNFRSF11B*) (log2-fold change = −2.95), and Osteonectin (*SPARC*) (log2-fold change = −1.17). ECM components related to vessel calcification were also down-regulated during space flight, *COL2A1* (log2-fold change = −3.37) and Versican (*VCAN*) (log2-fold change = −1.46). Proteins involved in both promotion and inhibition of calcium deposition were down-regulated, Alkaline Phosphatase (*ALPL*) (log2-fold change = −2.41) and Matrix Gla Protein (*MGP*) (log2-fold change = −2.06) respectively. The primary transcription factor associated with the osteogenic phenotype, *RUNX2*, did not experience a substantial transcriptional change (log2-fold change = 0.45). Two members of the Wnt family of transcription factors were up-regulated, *WNT3* (log2-fold change = 1.04) and *WNT16* (log2-fold change = 3.81). The expression of these transcription factors has been shown to lead to an osteogenic phenotype while preventing contractile polarization^26^.

### Pathway analysis

**Fig. 4:**
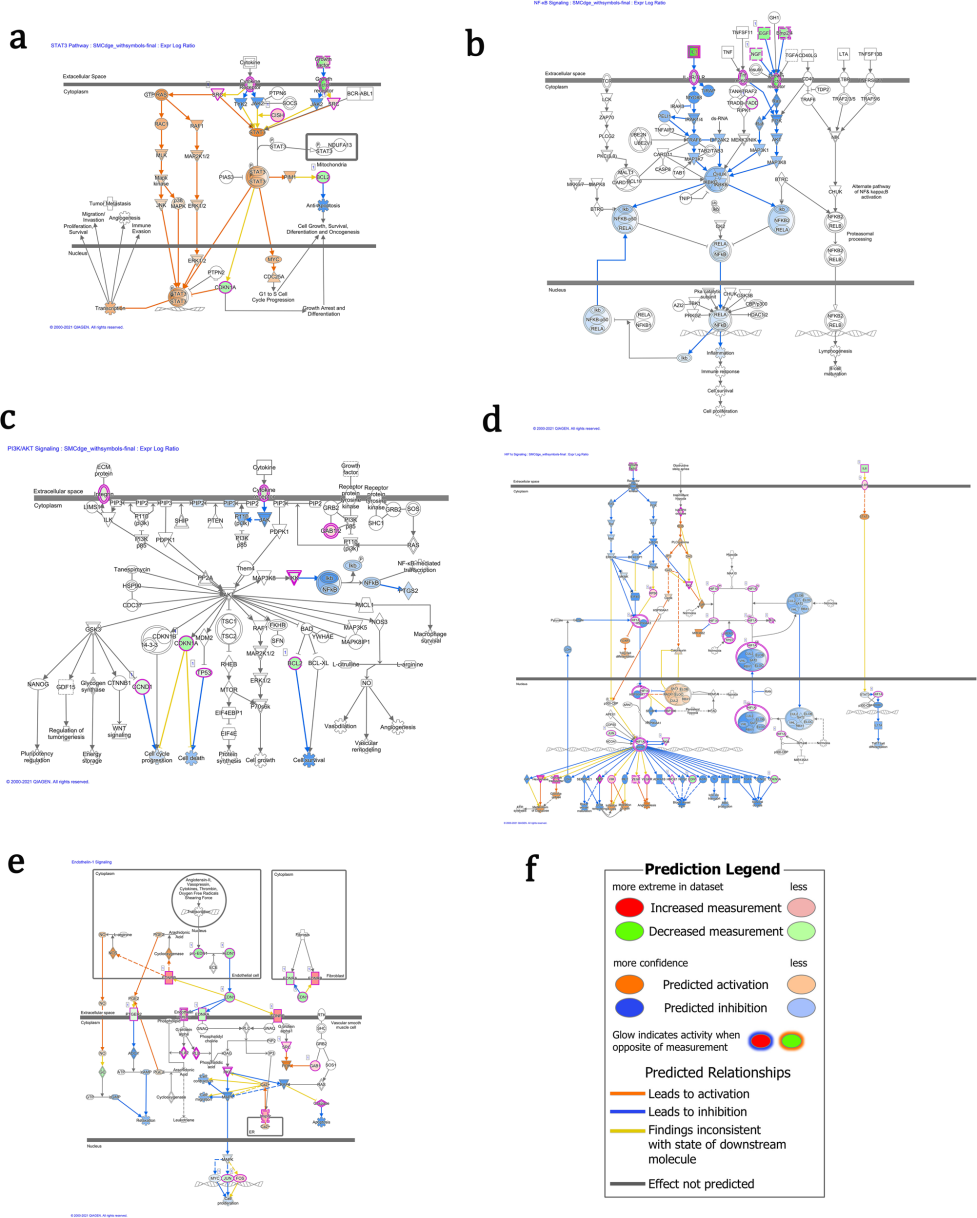
Key canonical pathways affected by space flight. Canonical pathways identified with a right-tailed Fisher’s Exact Test (*p*-value ≤ 0.05). The DEGs were down-regulated as indicated by the green color and up-regulated as indicated by the orange color. **a** STAT3 pathway, **b** HIF1α signaling, **c** NF-κB signaling, **d** PI3K/AKT signaling, **e** endothelin-1 signaling, **f** Figure Legend.

**Fig. 5:**
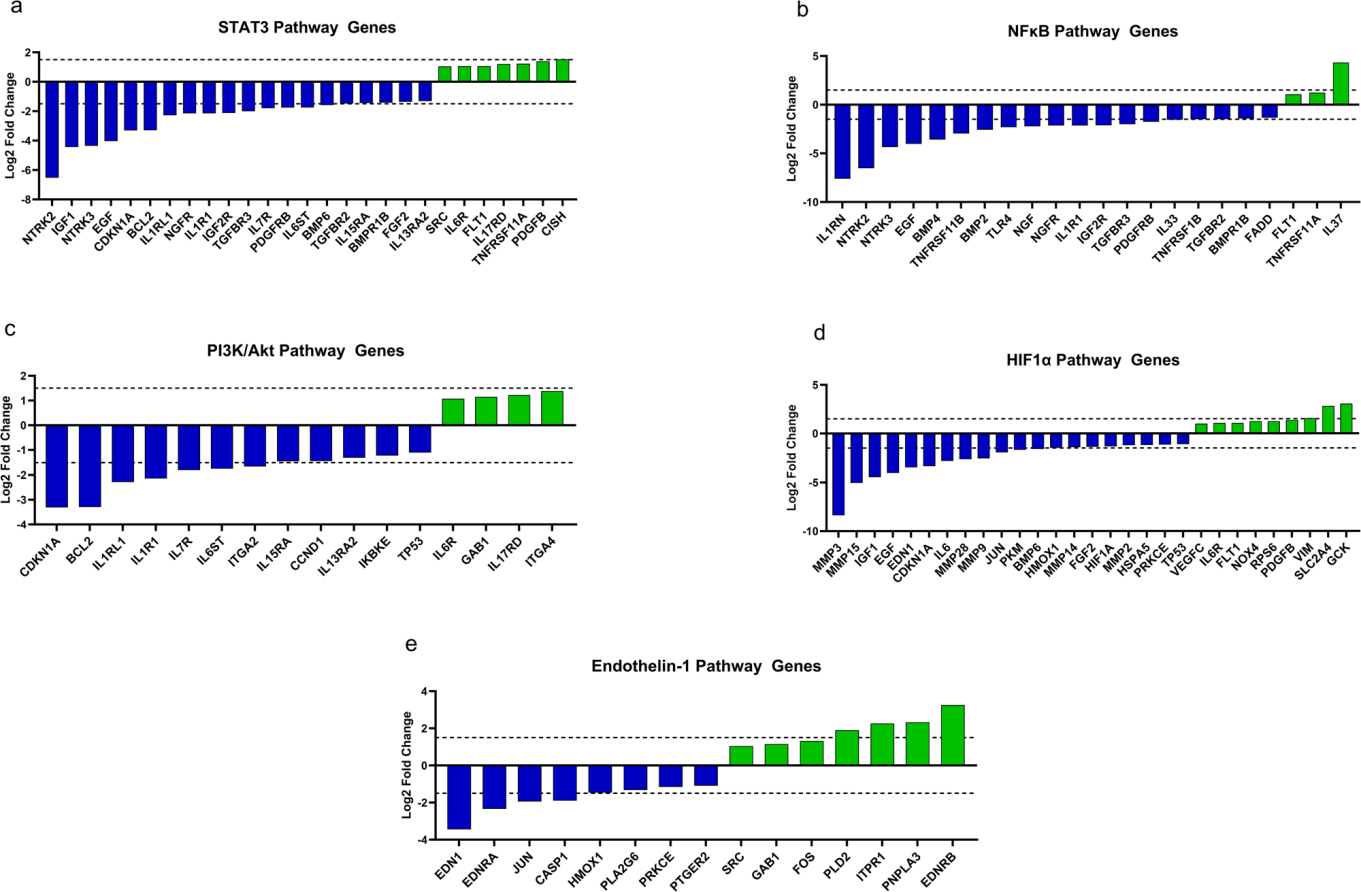
Fold change of pathway relevant transcripts. Log2-fold change of pathway relevant transcripts within the **a** STAT3 pathway, **b** NF-κB signaling, **c** PI3K/AKT signaling, **d** HIF1α signaling, **e** endothelin-1 signaling. Dashed line indicated a fold change of ±1.5.

The STAT3 signaling pathway is a ubiquitous pathway governing general cell functions like apoptosis, proliferation, and differentiation. Additionally, the STAT3 protein complex has been implicated in the phenotypic switching process of VSMCs and its expression is inversely related to the production of contractile apparatus components^27^. The IPA software predicted a general increase in the activation of a majority of the midstream pathway components, with a subsequent predicted increase in STAT3 transcription effects such as proliferation, survival, and angiogenesis (Fig. 4a). Despite the predicted increase in STAT3 activity, several relevant receptors were observed to be down-regulated during spaceflight including neurotrophic receptor tyrosine kinase 2 and 3 (*NTRK2/*3) (log2-fold change = −6.53, −4.35), interleukin-1 receptor type 1 (*IL1R1*) (log2-fold change = −2.14), insulin-like growth factor 2 receptor (*IGF2R*) (log2-fold change = −2.12), platelet derived growth factor receptor beta (*PDGFRB*) (log2-fold change = −1.76), and transforming growth factor beta receptor 2 (*TGFBR2*) (log2-fold change = −1.48). Log2-fold changes of *STAT3* transcripts are found in Fig. 5a. The STAT3 transcription factor underwent no significant transcriptional alteration. Notably, the gene BCL Apoptosis Regulator (Bcl-2) (*BCL2*) displayed decreased expression (log2-fold change = −3.29) while under microgravity followed by a predicted decrease in anti-apoptotic function (Fig. 4a). This presents a potential discrepancy as the predicted activation of STAT3 transcriptional activity could result in enhanced cell survival, while downregulation of Bcl-2 could indicate inhibition of anti-apoptotic activity^28^. Additionally, survivin (*BIRC5*), another inhibitor of apoptosis related to the STAT3 pathway, was up-regulated during spaceflight (log2-fold change = 3.29).

Down-regulated transcripts *IL1R1*, *NTRK2/3*, *PDGFBR*, and *TGFBR2* from the STAT3 pathway also overlap NFkB signaling. *TLR4* (log2-fold change = −2.32) and *TNFRSF11B* (log2-fold change = −2.95) were also down-regulated. IPA software predicted inhibition of several downstream proteins that rely on these receptors for activation. Subsequently, IPA predicted a slight inhibition of VSMC induced inflammation (Fig. 4b). This is further supported by the observed upregulation of *IL37* (log2-fold change = 4.33), which serves as an anti-inflammatory cytokine regulating both innate and adaptive immune responses^29,30^, and down-regulation of the pro-inflammatory *IL33* (log2-fold change = −1.54) and *IL6* (log2-fold change = −2.80). Additionally, *CCL2*, a chemokine involved in monocyte recruitment during inflammation^31,32^ was down-regulated (log2-fold change = −2.72), as well as cell surface molecules that recruit circulating immune cells like *VCAM1* (log2-fold change −4.50) and E-selectin (*SELE*) (log2-fold change = −4.85). Log2-fold changes of NFκB transcripts are found in Fig. 5b. Overall, it appears inflammatory processes related to the NFkB pathway were suppressed in VSMC during spaceflight.

PI3K/AKT signaling promotes cellular proliferation and produces important anti-apoptotic effects. Specific to VSMCs, PI3K/AKT signaling has been implicated in cytoskeletal rearrangement, phenotypic switching, and the progression of atherosclerotic lesion formation^33–35^. Our data shows a general decrease in expression of the cell surface receptors associated with PI3K activation (Fig. 4c). These include interleukin receptors for IL-1 (*IL1R1*) (log2-fold change = −2.14), IL-7 (*IL7R*) (log2-fold change = −1.80), IL-13 (*IL13RA2*) (log2-fold change = −1.31), and IL-15 (*IL15RA*) (log2-fold change = −1.44), as well as integrin subunit alpha 2 (*ITGA2*) (log2-fold change = −1.66). (Fig. 5c) Conversely, the IL-6 receptor (*IL6R*) (log2-fold change = 1.07) and integrin subunit alpha 4 (*ITGA4*) (log2-fold change = 1.38) were upregulated. Additionally, cyclin D1 (*CCND1*) expression was also decreased (log2-fold change = −1.44). As such, IPA software predicted inhibition of cell cycle progression and survival functions as expressed through the PI3K/AKT pathway (Fig. 4c). Transcription of the PI3K inhibitor (*PIK3IP1*) was enhanced (log2-fold change = 2.02) further supporting this prediction.

We also observed a modest decrease in Hypoxia Inducible Factor 1 Subunit Alpha (*HIF1A*) transcription expression (log2-fold change = −1.33). Conversely, IPA predicted an increase in the expression of several downstream components of the hypoxia induced activation pathway (Fig. 4d), potentially leading to an increased cellular sensitivity to hypoxic conditions. The significant downregulation of MMPs, particularly *MMP3* and *MMP15* as mentioned previously, lessen the potential of a synthetic phenotypic switch as synthetic VSMCs are highly migratory and utilize MMPs to remodel the surrounding extracellular matrix^36^. As such, the IPA software predicted an inhibition of ECM remodeling (Fig. 4d). Additionally, Osteopontin (*SPP1*) was heavily down-regulated (log2-fold change = −7.38). Log2-fold changes of *HIF1A* transcripts are found in Fig. 5d. The VSMCs exposed to space flight appear to display down-regulation of products relevant to both the synthetic and contractile phenotypes. IPA software also predicted the inhibition of several post pathway effects including cell survival and blood vessel maturation (Fig. 4d).

ET-1 signaling is primarily mediated in VSMCs though two receptors: ET_A_ (*EDNRA*) and ET_B_ (*EDNRB*), with ET_A_ being the primary ET-1 receptor^37^. Activation of the ET_A_ receptor results in activation of MAPK signaling pathway allowing for regulation of cellular contraction and blood pressure^38^. During space flight, the VSMCs displayed a down-regulation of *EDNRA* (log2-fold change = −2.34) followed by observed down-regulation and predicted inhibition of several MAPK signaling components. IPA provided predictions anticipating a reduction in contractility, migration, and proliferation (Fig. 4e). In contrast, *EDNRB* was up-regulated (log2-fold change = 3.26). Several transcription factors involved in ET-1 signaling were impacted by spaceflight including *FOS* (log2-fold change = 1.33) and *JUN* (log2-fold change = −1.94). The associated transcription factor *MYC* was not significantly altered (log2-fold change = −0.46). ET-1 itself (*EDN1*) was down-regulated (log2-fold change = −3.44). Additionally, the atheroprotective protein *HMOX1* (log2-fold change = −1.47) and the pro-inflammatory *CASP1* (log2-fold change = −1.90) were down-regulated (Fig. 5e).

## Discussion

For this investigation, we cultured VSMCs on spherical microcarrier beads aboard the ISS to observe how the space environment affects the cellular transcriptome. The selection of 3-dimensional culture via microcarrier beads is fairly standard for space flight studies as well as ground based using the rotating wall vessel (RWV) as we did. It is unknown how our experimental design compares to other culture modalities, such as flat 2D, cell spheroids, or culture within a 3D organoid, matrix or scaffold. Work in this area may reveal additional insight into cell-cell communication processes in microgravity. This work, however, lays a solid foundation for future studies into human smooth muscle cells in the space environment.

Our RNA-Seq analysis revealed over 11,000 genes whose expression upon exposure to the space environment was significant (*p* ≤ 0.05) in comparison to the ground control. 57% of the genes were down-regulated with the remaining 43% being up-regulated to some degree. Gene ontology analysis with the DAVID and PANTHER databases showed a majority of DEGs were associated with extracellular processes and extracellular matrix interaction terms. Two of the most represented cellular component terms were extracellular region and extracellular space, which relate to components and processes that are present outside the plasma membrane and largely consist of ECM and secreted compounds. Molecular function contained several binding terms including heparin, extracellular matrix, and glycosaminoglycan binding, as well as extracellular matrix structural constituent. This term refers to products that support the integrity of the ECM. Analysis of the cellular component and molecular function parent terms show a large change in ECM genes related to both production and cellular adherence. Additionally, analysis of the biological process parent term showed enrichment of the cell adhesion, cell-cell adhesion, and homophilic cell adhesion via plasma membrane adhesion molecules GO terms. Other biological process terms corresponded to a wide array of cellular processes including general signal transduction and cell-cell signaling, cell division, positive regulation of cell migration, and angiogenesis. Overall, GO analysis of SMCs exposed to spaceflight showed significant enrichment of terms largely related to ECM function and binding, and several cellular processes associated with proliferation, migration, and angiogenesis. Analysis of upregulated genes resulted in the enrichment of a number of GO terms related to processes and components used during cell division. The population of downregulated genes were associated with the GO terms for cellular adhesion and signal transduction, as well as various binding functions including protein, calcium ion, heparin, and integrin binding. KEGG pathway and GAD disease annotations both included terms related to cancer, indicating potential oncogenic behavior of SMCs while in space, although further investigation will be needed.

VSMCs can perform a diverse range of functions by modulating their phenotypes, with the most researched being contractile, synthetic, and osteogenic. These phenotypes are often associated with the production of distinct markers that both aid in identification and function. Due to the limitations of assessing gene expression in space, a caveat of this ISS study is that RNA transcript levels do not always reflect similar changes at the protein level so further studies will be important to extend our transcriptome data to the proteomic level^39^. The contractile phenotype is characterized by the expression and assembly of the contractile apparatus, a molecular machine that allows these cells to contract and relax in response to mechanical and chemical cues. Many aspects of the contractile apparatus were down-regulated during spaceflight. Of the two primary components of VSMC contractile machinery, *ACTA2* was down-regulated while *MYH11* remained unchanged. *ACTA2*, along with *MYH11*, produce the contractile filaments used by VSMCs during mechanical contraction. Additionally, several scaffold proteins involved in organization of the contractile apparatus were down-regulated to some degree, such as filamin and actin related proteins (ARP2/3) which serve as scaffolds and cross-linkers. The general downregulation of the contractile apparatus during spaceflight may coincide with the observed decrease in vascular tone and the onset of cardiovascular deconditioning seen in astronauts onboard the ISS^8–11^.

A distinct marker has yet to be found for synthetic VSMCs, however they have been characterized by their downregulation of the contractile apparatus, production of migratory mediators, and secretion of ECM proteins^23,24^. As discussed previously, several components of the contractile apparatus had been down-regulated during spaceflight. While this may lend credence to a synthetic phenotype, many migratory proteins were significantly down-regulated as well. Most notable of these include the MMP family which displayed a nearly ubiquitous reduction in transcription, of which MMP3 experienced one of the largest downregulations in our dataset. A reduction in MMP transcription would likely limit VSMC migratory potential as MMPs serve to cleave ECM proteins, aiding in cell motility. Additionally, synthetic VSMCs display enhanced ECM production to remodel the vasculature during injury.

The transcription of several ECM components were altered during spaceflight including select proteoglycans and collagen components that compose the primary elements of the vascular basement membrane, allowing for cellular adhesion and communication with the local environment. Prior studies have shown the presence of collagen IV to promote VSMC contractility and suppress phenotypic switching^40^. Virtually all other relevant collagen family gene members experienced little to no transcriptional change or were down-regulated. Transcription of collagen I, an important structural component of the vascular tunica as well as a mediator of vascular calcification^41^, remained largely unchanged. Collagen II, which has been shown to localize to sites of vascular calcification^21^ was down-regulated in our study. The proteoglycans lumican, decorin, and versican, which facilitate migration and the development of vascular diseases like atherosclerosis^42^, were strongly down-regulated. These transcriptional trends in ECM components during spaceflight indicate a potential inclination towards maintenance of the normal basement membrane and provide evidence of a non-synthetic phenotype.

Vascular calcification is a pathological condition often associated with chronic kidney disease, diabetes, and atherosclerosis^21,22^. Mineral deposition in the vasculature is primarily mediated by osteoblast-like VSMCs that have undergone a phenotypic switch towards a calcifying or osteogenic phenotype. Distinct markers for this phenotype are normally expressed by osteoblast during normal bone formation. RUNX2, an important transcription factor in osteogenic VSMC phenotypic polarization^21,43^, showed no significant change in transcription. BMP proteins initiate signaling through the Wnt transcription factor and serve as an early step in osteogenic differentiation^21^. The isoforms *BMP2, 4*, and *6* were all significantly down-regulated during spaceflight. Alkaline phosphatase was also down-regulated. This enzyme converts the calcification inhibitor inorganic pyrophosphate (PPi) to inorganic phosphate (Pi) as a key step in early vascular calcification^44^. Osteocalcin, the only osteogenic protein to be up-regulated, is present in areas of the vasculature undergoing vascular calcification and has been shown to promote osteogenic differentiation in VSMCs through the HIF1-α pathway^45^. Osteopontin, normally promoting bone resorption and inhibition of mineralization, is a multipurpose protein whose function is dependent on phosphorylation. The presence of osteopontin in the vasculature secreted by VSMCs is implicated in the progression of atherosclerotic plaque calcification^46,47^. Osteopontin was one of the most strongly down-regulated transcripts in our dataset. Several other osteogenic products were also down-regulated including osteoprogerin, versican, collagen type II, and matrix Gla protein. The downregulation of these genes suggests a lack of osteogenic phenotypic switching of VSMCs during spaceflight.

Cellular signaling pathways allow cells to respond to various external and internal stimuli. The associated signaling cascades lead to a wide variety of robust responses including survival and apoptosis regulation, DNA damage repair, angiogenesis, and phenotypic switching, among many others. These signaling pathways are often named in reference to the transcription factor that mediates the transcription of mRNA from DNA, which are subsequently translated into protein products. These proteins carry out the ensuing functions initiated by the original signaling event. STAT3 signaling regulates cellular proliferation, apoptosis, and VSMC phenotypic polarization^27^. This signaling pathway is initiated through ligand binding to several growth factor and cytokine receptors including IL-1R, FGFR, IGFR, NTRK, and EGFR^48^, transcripts of all were down-regulated during spaceflight. The STAT3 transcription factor did not experience a significant change in mRNA transcript production during spaceflight with a general trend toward downregulation of associated proteins. It is unclear how the anti-apoptotic function of STAT3 signaling was impacted by spaceflight as apoptosis mediators, Bcl-2 and survivin (*BIRC5)*, displayed divergent results with a decrease in *Bcl-2* transcription and an increase in survivin. Bcl-2 and survivin are both anti-apoptotic proteins, blocking the events that lead to programmed cell death. Upregulation of one and downregulation of the other makes it difficult to predict how apoptosis will progress via the STAT3 pathway. Further investigation will be required to elucidate the effects of spaceflight on VSMC proliferation and apoptosis mediated by STAT3 signaling.

The NFκB pathway is an important regulator for the progression of the cell cycle, proliferation, and pro-inflammatory gene expression^34,49^. The NFκB pathway has been implicated in the synthetic phenotypic switch due to its association with inflammatory cytokine production^50^. The NFκB dimer exists inactive in the cytosol and is activated by a large variety of signals including TNF cytokines, interleukins, and pathogen-associated molecular patters (PAMPs) such as lipopolysaccharide (LPS)^51^. These signals are transduced by their respective cell surface receptors including the TNF superfamily of receptors, interleukin receptors, and toll-like receptors (TLR)^34,52^. The products of NFκB transcription in VSMCs contribute to vascular wound healing, cell cycle progression, and immune cell recruitment^49^. Abnormal NFκB signaling is implicated in the development of atherosclerosis through the excessive activation of macrophages and smooth muscle proliferation^53,54^.

While four out of the five NFκB transcription complex subunits displayed a statistically significant change in transcription, they all fell below the log2-fold change cut off of ±1.50. However, many products of the NFκB pathway underwent a large degree of transcriptional regulation during spaceflight. Our data also showed a reduction NFκB inflammatory cytokine transcription IL-6, IL-33, and CCL2, as well as an upregulation of anti-inflammatory IL-37. Additionally, the cell surface proteins VCAM-1 and E-selectin involved in the activation and recruitment of immune cells experienced reduced transcription during spaceflight. Many receptors that initiate NFκB activation and transcription were also impacted by spaceflight, with a majority being down-regulated to a log2-fold change of −1.50. These results imply that spaceflight may result in a decrease in NFκB mediated inflammation in VSMCs.

Signaling via the PI3K/AKT pathway allows cells to regulate survival, proliferation, metabolism, and angiogenic processes. This signaling pathway does not lead to transcription, but rather the activation or suppression of proteins present in the cytosol though phosphorylation by the AKT protein kinase^55^. During spaceflight, none of the three AKT isoforms showed a significant change in transcription. Similarly, a majority of the PI3K complex components remained transcriptionally unchanged, either statistically or falling under the fold change threshold. Notably, the PI3K Interacting Protein 1 (*PIK3IP1*) was up-regulated. This protein binds to the catalytic domain of the PI3K p110 subunit to suppress its kinase activity^56^. These findings suggest the potential for a decrease in PI3K/AKT pathway activity during spaceflight. A reduction in migratory and angiogenic potential through the PI3K/AKT pathway may coincide with impaired wound healing seen in astronauts^57,58^.

Hypoxia-inducible factor 1α (HIF1α) expression has been shown to correlate with phenotypic polarization in vascular smooth muscle cells. Through its function as a transcription factor, VSMCs can be coaxed into a synthetic phenotype or display calcification and osteopontin production^59,60^. These functions have been implicated as a mechanism for the vessel stiffening observed in atherosclerosis and heart disease^61^. HIF1α pathway analysis suggests a complex response to space flight involving the downregulation of both synthetic and contractile related proteins (MMPs + osteopontin, *ACTA2*). Several MMP transcripts were significantly down-regulated during spaceflight with the most significant being MMP3 and MMP15. A reduction in the ability of VSMCs to polarize to either a contractile or synthetic phenotype could hinder the response from VSMCs to events like hyper/hypotension or vascular injury.

Signaling by ET-1 is integral to the normal function of the vasculature. Acting as a potent vasoconstrictor, ET-1 induces contraction of VSMCs as well as proliferation, migration, and ECM remodeling^37^. Of the two primary ET-1 signaling receptors, *EDNRA* was down-regulated while EDNRB was up-regulated. Previous studies suggest that *EDNRA* promotes signaling through the ET pathway while *EDRNB* allows VSMCs to sequester the ET-1 cytokine, providing a method for ET-1 clearance and negative regulation of the signaling pathway^62,63^. The contrasting transcriptional changes of these receptors indicate a shift towards the increased ET-1 sequestration in VSMCs during spaceflight.

ET-1 signaling provides the impetus for Protein Kinase C (PKC) activity, which leads to the upregulation of proliferative transcription products by the function of several transcription factors including c-Jun, c-Fos, and c-Myc^28,64^. Of which, c-Jun was down-regulated, c-Fos was up-regulated, and c-Myc experienced minor downregulation. Additionally, ET-1 signal transmission is intimately connected to the calcium dependent signaling involving the ingress of Ca^+2^ into the cytoplasm. Release of stored calcium from the endoplasmic reticulum is required for direct regulation of VSMC contractility. The receptor *ITPR1* responsible for calcium release was up-regulated during spaceflight, however IPA software predicted an overall inhibition of cellular contractility, likely due to the action of other factors such as the upregulation of calponin (*CNN1*), which inhibits myosin ATPase and subsequent contractile ability.

Overall, the results of our transcriptomic analysis of VSMCs post spaceflight suggest a reduction in contractility and provide little evidence of a phenotypic switch to either a synthetic or osteogenic phenotype. A majority of transcripts in the selected pathways, and in the transcriptome as a whole, experienced some level of downregulation. This may hint to a potential mechanism that leads to broad transcriptional inhibition in VSMCs initiated by exposure to spaceflight. This study aimed to highlight the large-scale transcriptional alteration that occurs at the cellular level when exposed to the space environment. This work joins a body of work into the effects of microgravity on smooth muscle cells and provides additional insight into human cells. While our study lasted 72 h on orbit we know there is some adaptation that occurs in space or in microgravity in other cell populations. Based upon other reports, we would expect there is also some adaptation by smooth muscle cells, however, the damaging effects from space radiation may have longer effects. We would hypothesize that if exposed to the space environment beyond 72 h the SMCs may adapt to microgravity with some cellular processes remining unchanged, however we would expect the genetic changes may be additive and will result in altered function of the vascular smooth muscle cells. The results presented in this study will benefit future investigations that probe the specific mechanisms by which cells alter their behavior in response to spaceflight.

## Methods

All the pre-flight experimental parameters and experimental details were optimized prior to flight during the experiment verification tests (EVT). Additional details of these optimizations are provided in the Supplemental Methods section.

### Cell culture

**Fig. 6:**
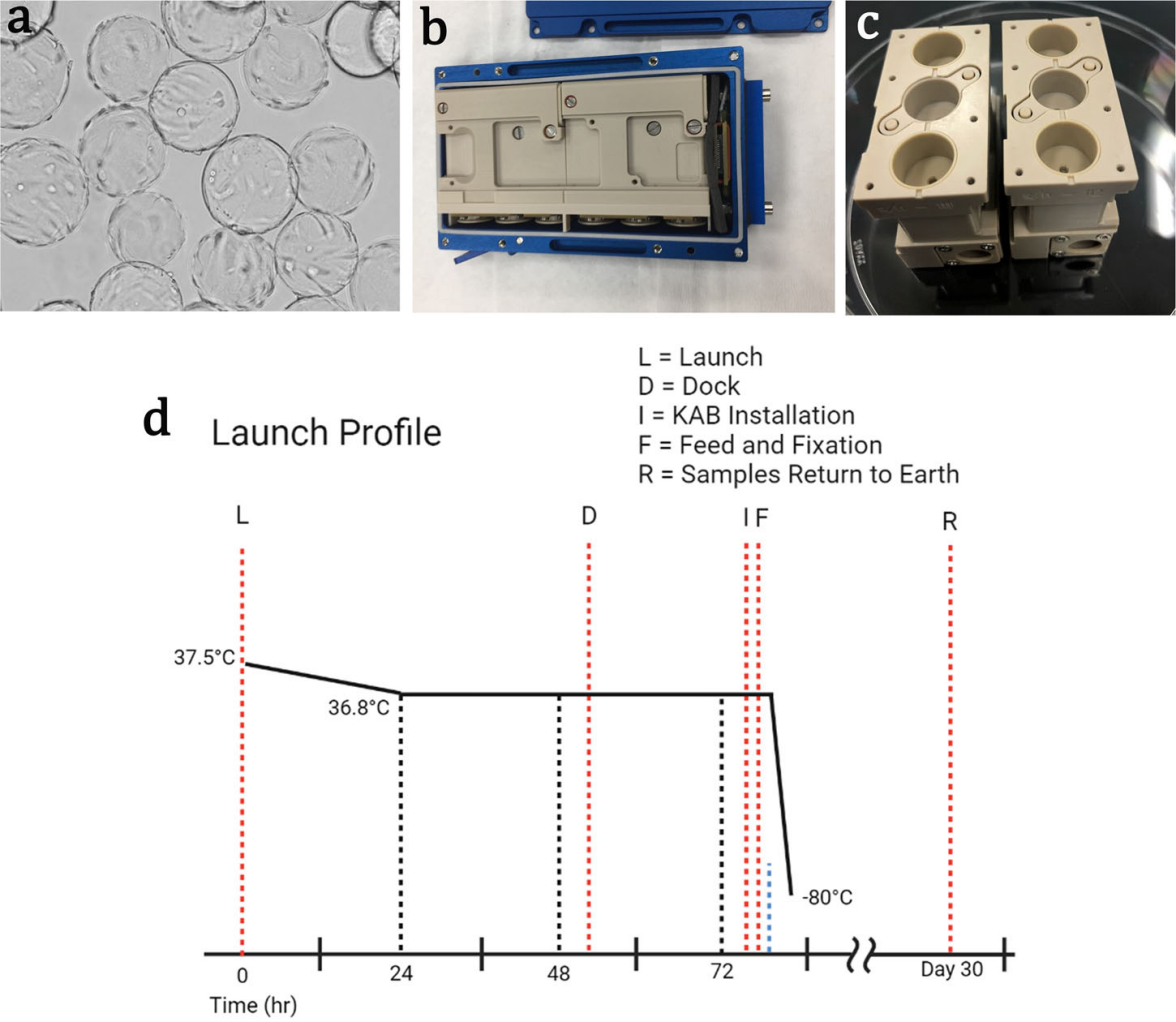
Experimental design parameters used in study. **a** HASMC attached to microcarrier beads. **b**, **c** Kayser Italia KEU-RO Experimental hardware. **b** assembled KEU-RO within its KIC housing chamber, **c** 2 halves of the brick each containing 3 cell culture chambers, for a total of 6 separate cylinders per brick. **d** Launch and experimental profile. Schematic showing the recorded hardware/sample temperatures and key time points for the main events occurring between the launch of the Space-X dragon capsule “L”, to the docking of the capsule to the ISS “D”, followed by the installation of the KEU-RO units into the incubator space “I”, the units then received refreshed media and were fixed in RNAlater “F”. The cassettes of fixed samples remained stored at −80 °C until being returned to earth after 30 days.

Human aortic smooth muscle cells (HASMC) and smooth muscle cell growth media (SmGM) was purchased from Cell Applications (San Diego, CA). The growth media consists of basal media supplemented with 5% fetal bovine serum, recombinant insulin, epidermal growth factor (EGF), basic fibroblast growth factor (bFGF), insulin-like growth factor (R3IGF), ascorbic acid, pen/strep, and amphotericin B, and was used for all pre-flight, flight, and ground-based cultures. HASMC are adherent cells, as such, in all space flight and ground-based cultures the three technical replicates of HASMC were seeded onto fibronectin coated (25 µg ml^−1^) cytodex-1 microcarrier beads (1 mg ml^−1^) (Sigma, St. Louis, MO) at a density of 10 cells per microcarrier bead in an ultra-low attachment plate (Corning, Corning, NY), and allowed to attach overnight at 37 °C, 5% CO2 in a humidified chamber (Fig. 6a). Additional details regarding optimization of these parameters is provided in the supplemental data section. Cells were cultured on microcarrier beads an additional three days in normal gravity to allow for proliferation and acclimation to the beads prior to loading into the flight hardware cell culture chambers. Prior to loading the HASMCs into the KEU-RO cell culture chambers, media was changed to fresh SmGM.

### Flight hardware and misson profile

The Bioscience-3 flight hardware set consisted of 6 KAB controller interface containers (KIC), each housing one Kayser experimental unit (KEU-RO) developed by Kayser Italia (Livorno, Italy) (Fig. 6b). Each KEU-RO was constructed from a biologically compatible plastic, polyetheretherketone (PEEK) consisting of two culture chamber housings (CCH). Each CCH contains two separate cell culture chambers with two separate independent fluid systems that function via a piston system to deliver SmGM-2 or RNAlater fixative (Invitrogen, Waltham, MA, USA) (Fig. 6c) allowing each complete KEU-RO assembly to hold four separate experimental samples. Three KEU-RO cell culture chambers housing three replicate populations of smooth muscle cells were sent to space. Once aboard the International Space Station (ISS), the KEU-RO were inserted into the Space Technology and Advanced Research Systems (STaARS) incubator where temperature was actively maintained at 37 °C. The three chambers were cultured in microgravity for three days, including both the time in orbit and installation in the International Space Station (ISS). The KEU-RO are capable of automated media addition to the cell culture chambers as well as the addition of RNAlater fixative at the experimental end point on a pre-defined timeline (Fig. 6d). Once fixed, the KICs containing the KEU-RO were moved and maintained at −80 °C until return to Earth. The ground control experiments were also conducted in the KEU-RO hardware units and were manually activated for media refresh and cell fixation using the same experimental protocol and temperature conditions as the space flight samples.

### Falcon Dragon spaceflight with Space-X CRS-17

SpaceX CRS-17 was a Commercial Resupply Services (CRS) mission to the ISS. The SpaceX Falcon 9 rocket was launched along with the Dragon capsule from Space Launch Complex 40 at Cape Canaveral Air Force Station in Florida, USA. The CRS-17 delivered more than 5500 pounds of research, supplies, equipment, and hardware to the ISS, including the KEU-RO containing human HASMCs. Approximately 48 h after launch, the dragon capsule was docked to the ISS, and approximately 24 h later, the KEU-RO were installed in the STaARS-1 incubator space for active temperature control at 37 °C. While in flight to the ISS and in orbit prior to dock with the ISS, the KEU-RO temperatures were passively maintained and monitored in real-time. During ascent the temperatures held steady at 37 °C ± 0.5 °C until installation on board the ISS. Once installed in STaARS-1 incubator space aboard the ISS, the hardware temperature was maintained at 37 °C ± 1 °C. The samples from our study spent a total of 3 days in microgravity before being stored frozen, and a total of ~30 days aboard the International Space Station before return to earth.

### Cell fixation and RNA isolation

Upon insertion of the KEU-RO/KIC into the STAaRS-1 incubator, the KEU-RO were automatically activated to add 200 μl of refresh media to all of the units’ cell culture chambers. At 3 days in space, the units were fixed by automated injection of 900 μL RNAlater directly into the cell culture housing chambers (CCH), and transferred to −80 °C within 90 min of fixation. Upon return to earth, KIC units were thawed on ice and samples extracted from each KEU-RO. Total RNA was extracted from the SMCs preserved in RNAlater with the RNeasy RNA Extraction kit (QIAGEN, Germantown, MD, USA) in conjunction with QIAshredders columns (QIAGEN). Samples were DNase treated (Turbo DNase, Invitrogen, Waltham, MA) and quantitated with Quant-iT RiboGreen RNA assay (Invitrogen).

### RNA extraction, library construction and sequencing

Total RNA quality was assessed using a Fragment Analyzer. Poly(A) selected cDNA libraries were prepared (NEBNext UltraII Directional RNA Library Prep kit, NEB, Ipswich, MA), following manufacturers recommendations. SMC libraries were constructed by using 100 ng total RNA. Sequencing was performed on the NextSeq500 Illumina platform. FastQC^65^ was used to quality control on raw data and the reads acquired were cleaned up with the cutadapt program^66^ to trim off sequencing adapters and low-quality bases with a quality phred-like score <20. Differential gene expression studies used DESeq2^67^ (ver. 1.6.3). Genes with −1.5 ≤ log2-fold change ≤ 1.5 in expression and an adjusted *p* ≤ 0.05 were classified as significantly differentially expressed. RNA-seq data sets are deposited in NASA’s GeneLab Open Science Data Repository (OSDR)^68^.

### Pathway analysis and gene ontology (GO)

The network and pathway behaviors of spaceflight and ground control cultured smooth muscle cells based upon the differentially expressed genes we analyzed using the Ingenuity Pathway Analysis software (IPA, QIAGEN). In our analysis, a *p*-value of *p* ≤ 0.05 and a False Discovery Rate (FDR) or padj ≤ 0.05 were considered statistically significant. We restricted our analysis with IPA to human species with no other selection criterion. Differentially expressed genes from the RNA-seq analysis were filtered with a log2-fold change of ±1.50 and Benjamini–Hochberg’s false discovery rate at ≤0.05 and analyzed based on experimental evidence in the IPA Knowledge Base. In the core analysis of IPA, significantly enriched canonical pathways were identified with a right-tailed Fisher’s Exact Test and a z-score of ±2 was considered significant. The canonical pathway tool was used to identify the top canonical pathways, biological functions and networks associated with the genes differentially expressed between compared conditions. The right-tailed Fisher’s exact test was performed to calculate the *p*-value for measuring the overlap of observed and predicted regulated gene sets, and a z-score assessing the match of observed and predicted up/down regulation patterns.

The Database for Annotation, Visualization, and Integrated Discovery (DAVID) (v2023q1) Bioinformatics Resource^69,70^, available at https://david.ncifcrf.gov/home.jsp, was used for Gene Ontology (GO), Kyoto Encyclopedia of Genes and Genomes (KEGG) pathway (release 104.1), and Genetic Association Database (GAD) disease classification analysis (retired GAD database 2014). Differentially expressed genes with padj ≤0.05, −1.50 ≤ log 2-fold change ≤ 1.50, and a base mean ≥20 were uploaded to DAVID. 2616 DAVID IDs were recognized and grouped according to biological process, cellular component, molecular function, KEGG pathway, and GAD disease classification. GO analysis was supported by a comparative analysis with the PANTHER knowledgebase (v. 17.0). Annotations with a False Discovery Rate (FDR) ≤ 0.05 were treated as statistically significant. An additional analysis was performed by separating the upregulated and downregulated genes into distinct populations. The upregulated gene analysis resulted in 1127 genes recognized by DAVID and the downregulated gene analysis had 1473 associated DAVID IDs.

### Reporting summary

Further information on research design is available in the Nature Research Reporting Summary linked to this article.

### Supplementary information

Supplemental Data

Reporting Summary

## Data Availability

Data and Meta Data available upon request. RNA-seq data sets are deposited in NASA’s GeneLab Open Science Data Repository (OSDR) under OSDR accession number OSD-635 and 10.26030/8n5a-q187.
