# Supplementary material for: Spaceflight effects on human vascular smooth muscle cell phenotype and function
Source: NPJ Microgravity. 2024 Mar 28;10:41. doi: 10.1038/s41526-024-00380-w (PMC10979029; doi:10.1038/s41526-024-00380-w)
Supplement: Supplementary file 1 — Supplemental Data [file 41526_2024_380_MOESM1_ESM.pdf]

Spaceflight effects on human vascular smooth muscle cell phenotype and function

Marina M. Scotti[a], Brandon K. Wilson[b], Jodi L. Bubenik[c], Fahong Yu[d], Maurice S. Swanson[c], and Josephine B. Allen[a*]

a Department of Materials Science and Engineering, University of Florida

b Department of Biomedical Engineering, University of Florida

c Department of Molecular Genetics and Microbiology, Center for NeuroGenetics, University of Florida,

d Interdisciplinary Center for Biotechnology Research, University of Florida

* Corresponding Author

<u>Corresponding Author</u>
Josephine Allen, Ph.D.
Department of Materials Science and Engineering
University of Florida
100 Rhines Hall
Gainesville, FL 32611

Keywords: microgravity, RNA-Seq, transcriptomics, contractile, proliferation

Supplemental Data

A. Supplemental Methods:

Here we provide some additional details to describe the pre-flight optimizations and experimental parameters that were selected for this study. Prior to space flight we optimized the following parameters:

Number of cells/bead and beads/chamber: To ensure cell viability within the hardware chambers for an extended period of time, we systematically varied the cell seeding density per bead (~1, 2, 3, 5, and 10 cells/bead) as well as varied the number of beads that would be inserted into the chamber (4.125 mg beads, 1.0 mg beads, 0.75 mg beads). Our goal was to balance obtaining enough genetic material for subsequent analysis while also choosing conditions that result in increased cell viability. Visually, cell viability was assessed via Live/dead staining kit. For our study we selected to use 10cells/bead and to insert 1mg of cell seeded beads into the hardware chambers.

Hardware Biochemical Compatibility Test: Prior to flight the hardware material polyetheretherketone (PEEK) was tested biologically. Biological testing involved assessing the hardware material to the human vascular smooth muscle cells, in the planned complete flight media, and the preservative RNAlater. Once filled with each of the test conditions, the hardware material was incubated for what would be the flight duration, and it was found that the hardware did not cause any significant change in cell viability.

Equipment Verification Test (EVT): The work of the EVT focused on the verification of the hardware loading and experimental parameters in a mock flight set-up. The EVT was conducted using estimated parameters such as timing and temperature of hardware loading, incubation for up to 8.5 days, then fixation with RNA later, freezing at -80°C, followed by thawing of the samples, then the quantification of RNA yield. We conducted 3 separate EVT tests and optimized or validated the following parameters:

Seamless and speedy hardware loading – Out team trained on hardware assembly and cell loading extensively so that when ready for flight prep we would be able to get the cells into the unit and back into the incubator in a timely manner.

Validation of cell viability in the hardware – Our prior optimizations for cell density occurred in microfuge tubes, therefore it was necessary to validate that the cells remained viable within the hardware and following the extensive manipulations necessary for the KEURO loading. During our EVT experiments, we validated that the cells in the KEURO units appear attached and through day 8.5 within the EVT test.

RNA Quantity and Quality: We validated that the cell density and RNA later concentrations would yield usable quality of RNA to do our post-flight transcriptomics. Our results validated that a 1:1 ratio of cells in media to RNA later at 37°C was stable and the RNA remained intact for up to 4 hours. This information was incorporated into our flight parameters to ensure the hardware was moved to -8- storage within 4 hours. When the cell lysate RNA was run on an ethidium bromide gel, we show successful separation of 28s and 18s bands at the ratio of 2:1, indicating intact RNA devoid of degradation.

B. Supplemental Figures

Supplemental Figure 1 - The Cardiovascular Disease and Cardiovascular System Development and Function Associated IPA Networks

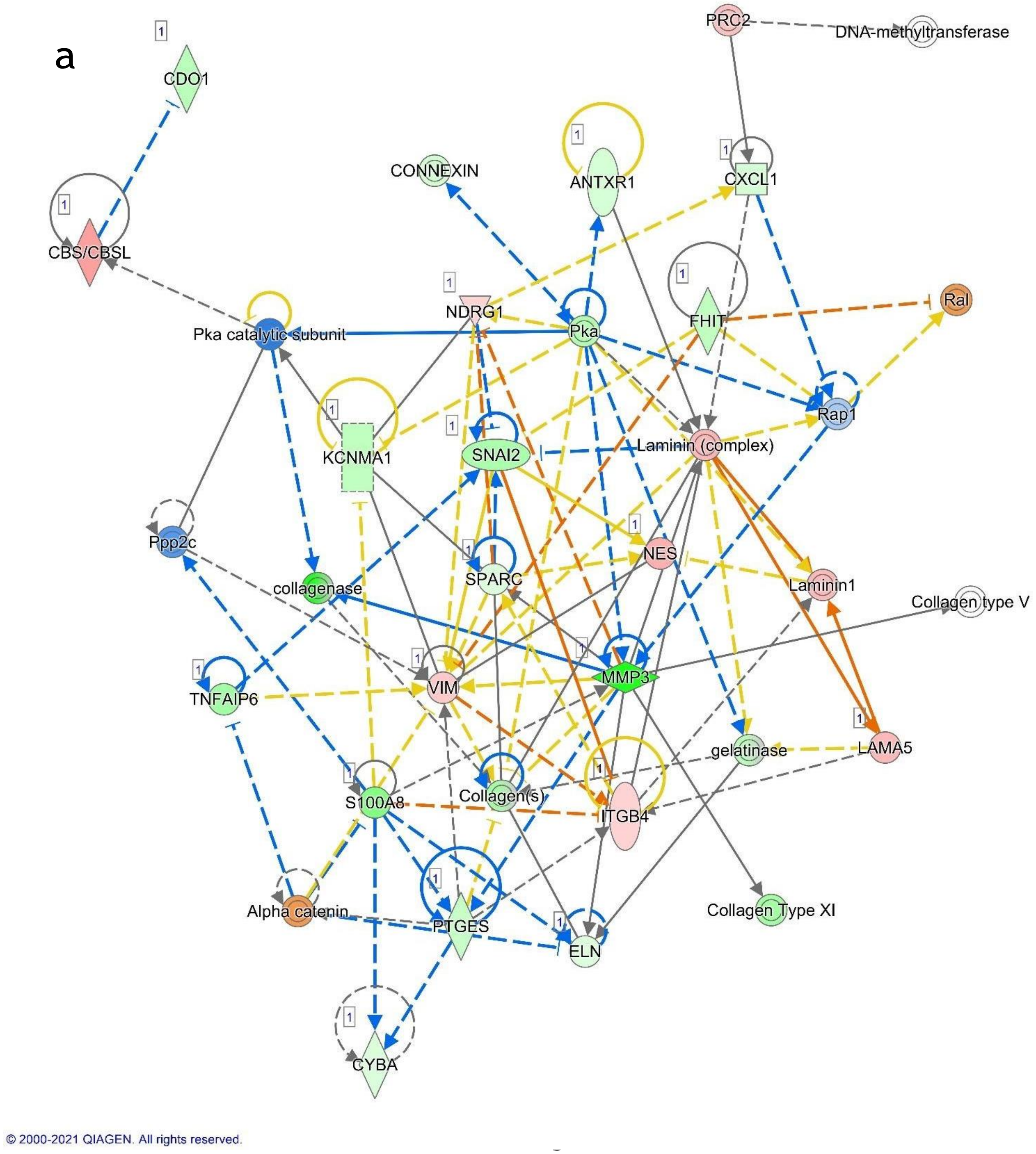

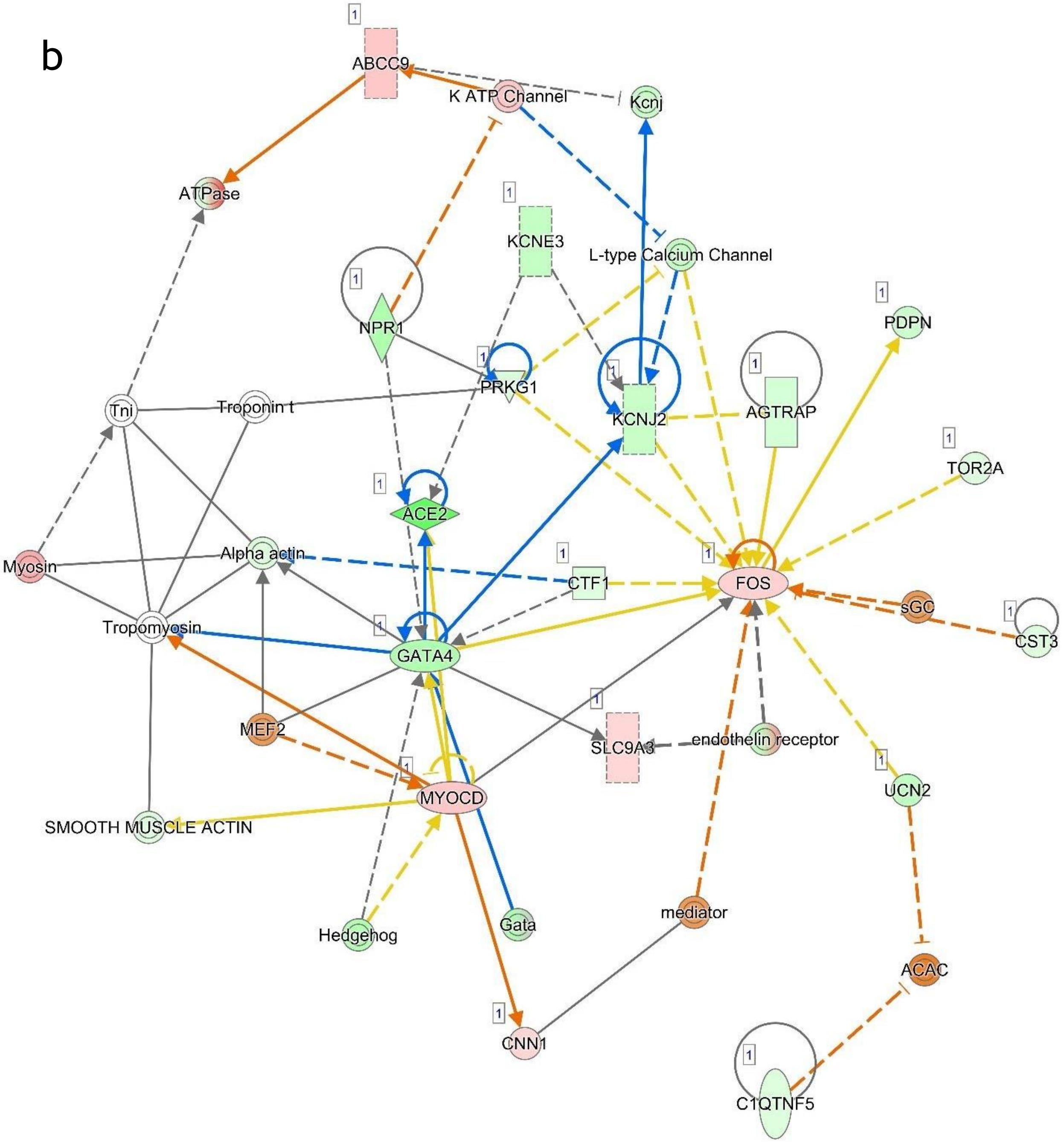

© 2000-2021 QIAGEN. All rights reserved.

C

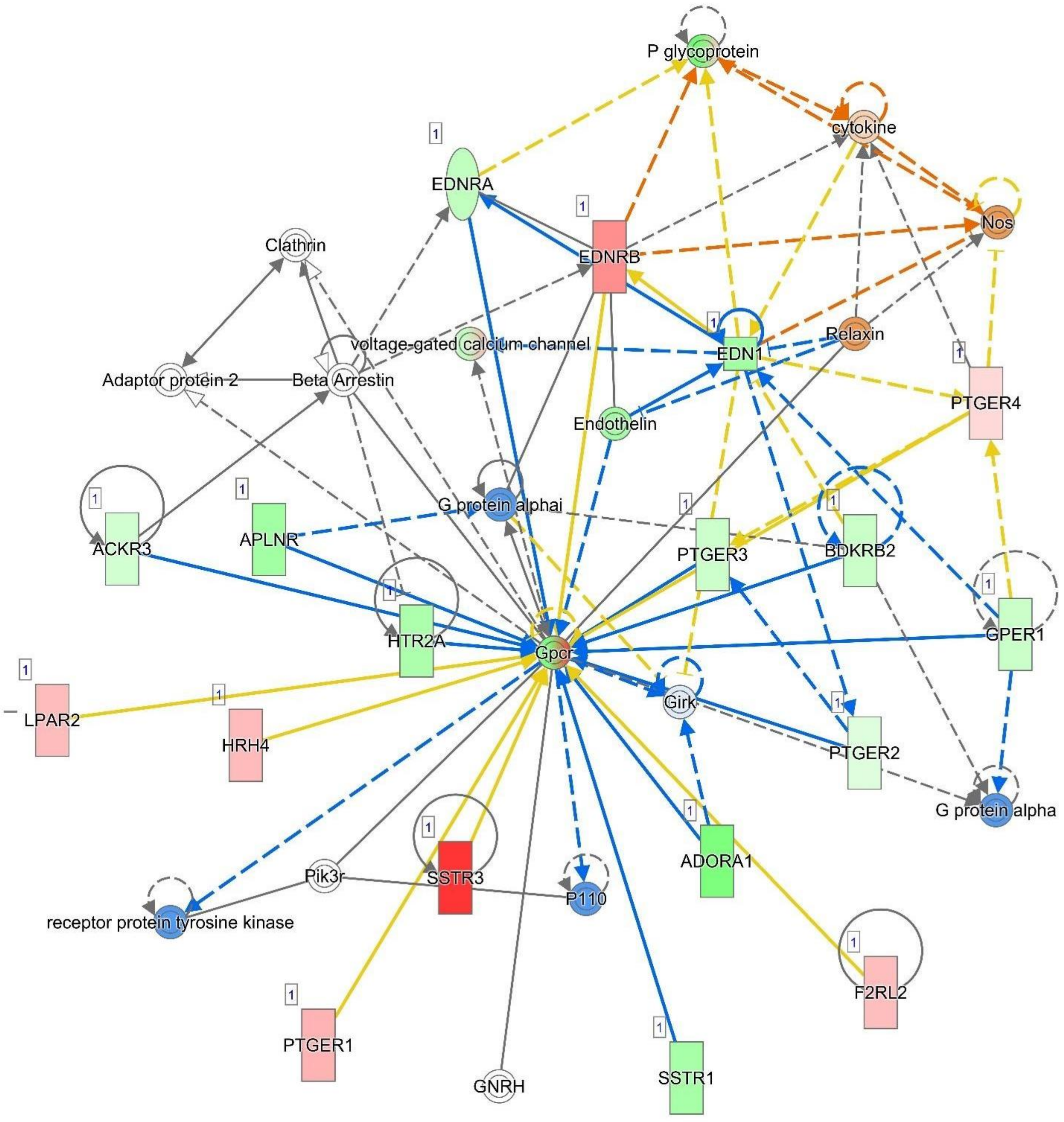

Supplementary Figure 1. The cardiovascular disease and cardiovascular system development and function associated IPA networks. **a**: cardiovascular system development and function, cellular movement, organismal development **b**: cardiovascular disease, cardiovascular system development and function, organismal development **c**: cardiovascular system development and function, cell-to-cell signaling and interaction, hematological system development and function. Genes that are upregulated and downregulated were displayed within red and green nodes, respectively. Solid and dashed lines between genes represent known direct and indirect gene interactions, respectively. The shapes of the nodes reflect the functional class of each gene product: transcriptional regulator (horizontal ellipse), transmembrane receptor (vertical ellipse), enzyme (vertical rhombus), cytokine/growth factor (square), kinase (inverted triangle) and complex/group/other (circle).

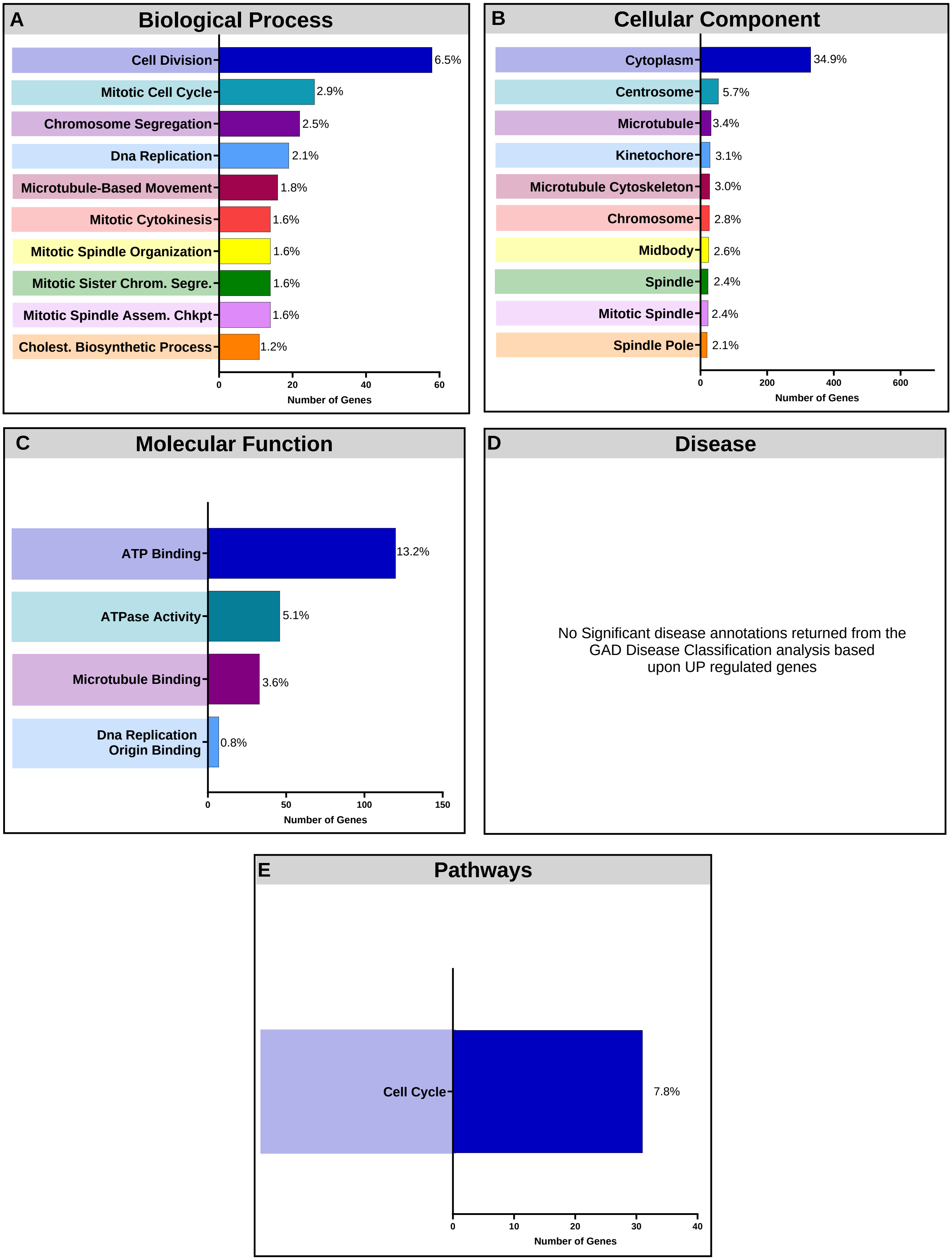
A
Biological Process
Cell Division
Mitotic Cell Cycle
Chromosome Segregation
Dna Replication
Microtubule-Based Movement
Mitotic Cytokinesis
Mitotic Spindle Organization
Mitotic Sister Chrom. Segre.
Mitotic Spindle Assem. Chkpt
Cholest. Biosynthetic Process
6.5%
2.9%
2.5%
2.1%
1.8%
1.6%
1.6%
1.6%
1.6%
1.2%
0
20
40
60
Number of Genes
B
Cellular Component
Cytoplasm
Centrosome
Microtubule
Kinetochore
Microtubule Cytoskeleton
Chromosome
Midbody
Spindle
Mitotic Spindle
Spindle Pole
34.9%
5.7%
3.4%
3.1%
3.0%
2.8%
2.6%
2.4%
2.4%
2.1%
0
200
400
600
Number of Genes
C
Molecular Function
ATP Binding
ATPase Activity
Microtubule Binding
Dna Replication
Origin Binding
13.2%
5.1%
3.6%
0.8%
0
50
100
150
Number of Genes
D
Disease
No Significant disease annotations returned from the
GAD Disease Classification analysis based
upon UP regulated genes
E
Pathways
Cell Cycle
7.8%
0
10
20
30
40
Number of Genes

Supplemental Figure 2. DAVID analysis of DEGs of p-adj ≤ 0.05, log2-fold change ≥ 1.5 (Up regulated), and base mean expression ≥ 20. 1,151 genes were uploaded with 1,127 recognized by DAVID. Percents indicate the ratio of genes related to the specific annotation to the total number of assigned genes for the analysis. The ten highest GO terms representing (**A**) the Biological Process annotation; (**B**) the Cellular Component annotation; and (**C**) the Molecular Function annotation. (**D**) Ten highest represented disease annotations returned from the GAD Disease Classification analysis. (**E**) Significant pathways returned from KEGG analysis. Terms with a False Discovery Rate (FDR) ≤ 0.05 were treated as statistically significant.

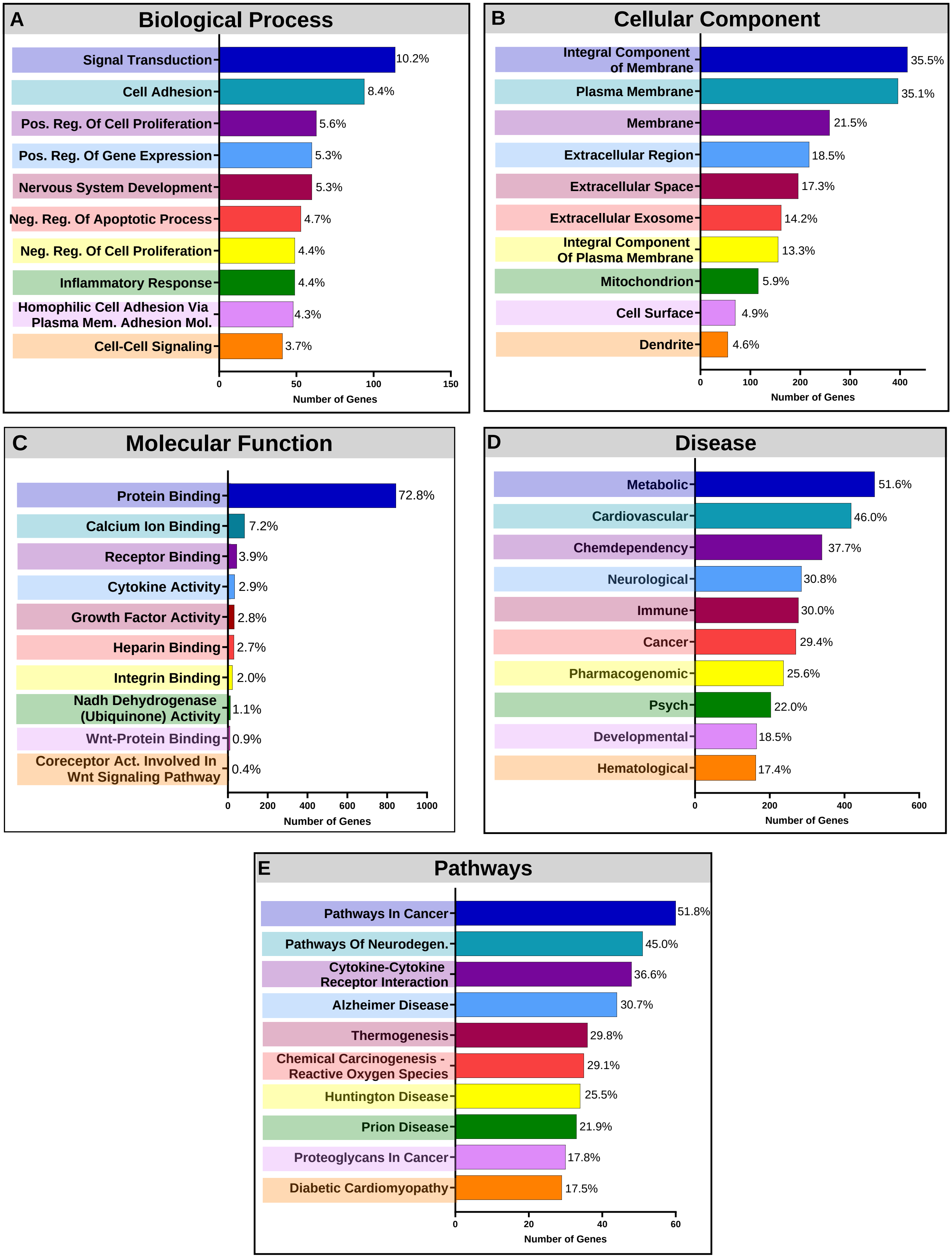

A
Biological Process
Signal Transduction
Cell Adhesion
Pos. Reg. Of Cell Proliferation
Pos. Reg. Of Gene Expression
Nervous System Development
Neg. Reg. Of Apoptotic Process
Neg. Reg. Of Cell Proliferation
Inflammatory Response
Homophilic Cell Adhesion Via Plasma Mem. Adhesion Mol.
Cell-Cell Signaling
10.2%
8.4%
5.6%
5.3%
5.3%
4.7%
4.4%
4.4%
4.3%
3.7%
0
50
100
150
Number of Genes
B
Cellular Component
Integral Component of Membrane
Plasma Membrane
Membrane
Extracellular Region
Extracellular Space
Extracellular Exosome
Integral Component Of Plasma Membrane
Mitochondrion
Cell Surface
Dendrite
35.5%
35.1%
21.5%
18.5%
17.3%
14.2%
13.3%
5.9%
4.9%
4.6%
0
100
200
300
400
Number of Genes
C
Molecular Function
Protein Binding
Calcium Ion Binding
Receptor Binding
Cytokine Activity
Growth Factor Activity
Heparin Binding
Integrin Binding
Nadh Dehydrogenase (Ubiquinone) Activity
Wnt-Protein Binding
Coreceptor Act. Involved In Wnt Signaling Pathway
72.8%
7.2%
3.9%
2.9%
2.8%
2.7%
2.0%
1.1%
0.9%
0.4%
0
200
400
600
800
1000
Number of Genes
D
Disease
Metabolic
Cardiovascular
Chemdependency
Neurological
Immune
Cancer
Pharmacogenomic
Psych
Developmental
Hematological
51.6%
46.0%
37.7%
30.8%
30.0%
29.4%
25.6%
22.0%
18.5%
17.4%
0
200
400
600
Number of Genes
E
Pathways
Pathways In Cancer
Pathways Of Neurodegen.
Cytokine-Cytokine Receptor Interaction
Alzheimer Disease
Thermogenesis
Chemical Carcinogenesis - Reactive Oxygen Species
Huntington Disease
Prion Disease
Proteoglycans In Cancer
Diabetic Cardiomyopathy
51.8%
45.0%
36.6%
30.7%
29.8%
29.1%
25.5%
21.9%
17.8%
17.5%
0
20
40
60
Number of Genes

Supplemental Figure 3. DAVID analysis of DEGs of p-adj ≤ 0.05, log2-fold change ≤ -1.5 (Down regulated), and base mean expression ≥ 20. 1,528 genes were uploaded with 1,473 recognized by DAVID. Percents indicate the ratio of genes related to the specific annotation to the total number of assigned genes for the analysis. The ten highest GO terms representing (**A**) the Biological Process annotation; (**B**) the Cellular Component annotation; and (**C**) the Molecular Function annotation. **(D**) Ten highest represented disease annotations returned from the GAD Disease Classification analysis. (**E**) Significant pathways returned from KEGG analysis. Terms with a False Discovery Rate (FDR) ≤ 0.05 were treated as statistically significant.

C. Supplemental Tables

Supplemental Table S1 – Full List of Canonical Pathways from IPA

Ingenuity Canonical Pathways	-log(p-value)	Ratio	z-score	Genes
**PTEN Signaling**	8.52	0.127	2	BCL2,BMPR1B,CCND1,CDKN1A,FLT1,IGF2R,IKBKE,ITGA2,ITGA4,NGFR,NTRK2,NTRK3,PDGFRB,TGFBR2,TGFBR3,TNFRSF11 A
**PPARα/RXRα Activation**	6.01	0.0842	2	ACVR2A,ACVR2B,CLOCK,FASN,IKBKE,IL1R1,IL1RL1,IL6,IRS1,JUN,PPARA,PPARGC1A,PRKAR2B,SMAD3,TGFBR2,TGFBR3
**Reelin Signaling in Neurons**	2.44	0.062	1.89	APOE,GRIN1,ITGA2,ITGA4,ITGB3,LRP8,SRC,VLDLR
**LXR/RXR Activation**	19.3	0.215	1.877	APOA4,APOC1,APOD,APOE,C3,CCL2,CD14,CLU,FASN,HMGCR,IL1R1,IL1RL1,IL1RN,IL33,IL37,IL6,MMP9,MSR1,NGFR,NR1H2,S100A8,SAA1,TLR4,TNFRSF11B,TNFRSF1B,VTN
**Estrogen-mediated S-phase Entry**	5.08	0.231	1.633	CCND1,CDK1,CDK2,CDKN1 A,E2F1,ESR2
**PPAR Signaling**	9.78	0.154	1.5	FOS,IKBKE,IL1R1,IL1RL1,IL1RN,IL33,IL37,JUN,NGFR,PDGFA,PDGFB,PDGFRB,PPARA,PPARGC1A,TNFRSF11B,TNFRSF1B
**Inhibition of Matrix Metalloproteases**	7.35	0.231	1.414	MMP14,MMP15,MMP2,MMP28,MMP3,MMP9,SDC1,TIMP1,TIMP4
**Insulin Receptor Signaling**	1.26	0.0429	1.342	GAB1,IRS1,PRKAR2B,SCNN1B,SCNN1 G,SLC2A4
**Cyclins and Cell Cycle Regulation**	5.44	0.123	1.265	CCND1,CCND2,CDK1,CDK2,CDKN1 A,CDKN2A,CDKN2B,CDKN2D,E2F1,TP53
**Non-Small Cell Lung Cancer Signaling**	4.94	0.123	1.134	CCND1,CDKN2A,E2F1,EGF,FHIT,ITPR1,RARB,RASSF1,TP53
**Melanoma Signaling**	2.56	0.1	1	CCND1,CDKN1A,CDKN2 A,E2F1,TP53
**Cell Cycle Regulation by BTG Family Proteins**	2.26	0.108	1	BTG2,CCND1,CDK2,E2F1
**Calcium-induced T Lymphocyte Apoptosis**	2.04	0.0758	1	ATP2A3,ITPR1,NR4A1,ORAI1,PRKCE
**SPINK1 Pancreatic Cancer Pathway**	1.54	0.0667	1	CTSB,F2RL1,SMAD3,TGFBR2

**Synaptogenesis Signaling Pathway**	1.38	0.0353	0.905	APOE,EFNB2,GRIN1,ITPR1,LRP8,NTRK2,PRKAR2B,PRKCE,SRC,STX1A,VLDLR
**Glioblastoma Multiforme Signaling**	6.09	0.0909	0.832	CCND1,CDK2,CDKN1A,CDKN2 A,E2F1,EGF,IGF1,ITPR1,NF1,PDGFA,PDGFB,PDGFRB,RND3,SRC,TP53
**Role of NANOG in Mammalian Embryonic Stem Cell Pluripotency**	5.46	0.101	0.816	BMP2,BMP4,BMP6,BMPR1B,GAB1,GATA4,IL6ST,LIF,POU5F1,SALL4,SMAD9,TP53
**Natural Killer Cell Signaling**	0.735	0.0305	0.816	COL2A1,COL3A1,HSPA5,IL12A,IL15,MICA
**PDGF Signaling**	3.58	0.093	0.707	CAV1,FOS,JUN,PDGFA,PDGFB,PDGFRB,SRC,STAT1
**eNOS Signaling**	3.55	0.0692	0.632	BDKRB2,CAV1,ESR2,FLT1,HSPA5,ITPR1,LPAR2,PRKAR2B,PRKCE,PRKG1,VEGFC
**Endocannabinoid Cancer Inhibition Pathway**	4.64	0.0839	0.577	CASP1,CCND1,CCND2,CDKN1 A,HIF1 A,MMP2,PRKAR2B,SNAI2,SPTLC2,SRC,VEGFC,VIM
**Ephrin Receptor Signaling**	3.67	0.0667	0.447	ANGPT1,CXCL12,EFNB2,EGF,FGF1,GRIN1,ITGA2,ITGA4,PDGFA,PDGFB,SRC,VEGFC
**VEGF Family Ligand-Receptor Interactions**	2.24	0.0714	0.447	FLT1,FOS,NRP1,PLA2G6,PRKCE,VEGFC
**Antioxidant Action of Vitamin C**	1.71	0.055	0.447	HMOX1,IKBKE,PLA2G6,PLD2,PNPLA3,SLC2 A4
**Synaptic Long Term Potentiation**	1.4	0.0465	0.447	CACNA1C,GRIN1,ITPR1,PRKAR2B,PRKCE,RAPGEF3
**PAK Signaling**	1.39	0.0515	0.447	ITGA2,ITGA4,PDGFA,PDGFB,PDGFRB
**Rac Signaling**	1.18	0.0446	0.447	CD44,ITGA2,ITGA4,JUN,NOX4
**Sphingosine-1-phosphate Signaling**	1.11	0.0427	0.447	CASP1,PDGFA,PDGFB,PDGFRB,RND3
**Endocannabinoid Neuronal Synapse Pathway**	0.987	0.0391	0.447	CACNA1C,CACNA1G,GRIN1,ITPR1,PRKAR2B
**Cdc42 Signaling**	0.652	0.0299	0.447	FOS,ITGA2,ITGA4,JUN,SRC
**Regulation of Cellular Mechanics by Calpain Protease**	3.59	0.108	0.378	CCND1,CDK1,CDK2,EGF,ITGA2,ITGA4,SRC
**Gαi Signaling**	2.52	0.064	0.378	ADORA1,APLNR,CAV1,NPR3,PR KAR2B,PTGER3,SRC,SSTR3
**Neuropathic Pain Signaling In Dorsal Horn Neurons**	2.46	0.0693	0.378	FOS,GRIN1,ITPR1,NTRK2,PRKAR2B,PRKCE,SRC

**p70S6K Signaling**	1.89	0.0543	0.378	EEF2,F2RL1,F2RL2,IRS1,PRKCE,RPS6,SRC
**Th1 Pathway**	3.23	0.0744	0.333	CD274,DLL1,DLL4,GATA3,IL12A,IL6,IL6R,IRF1,STAT1
**HGF Signaling**	5.78	0.108	0.302	CCND1,CDK2,CDKN1A,CDKN2 A,ELF3,FOS,GAB1,IL6,ITGA2,ITGA4,JUN,PRKCE
**Endothelin-1 Signaling**	5.38	0.0798	0.258	CASP1,EDN1,EDNRA,EDNRB,FOS,GAB1,HMOX1,ITPR1,JUN,PLA2G6,PLD2,PNPLA3,PRKCE,PTGER2,SRC
**T Cell Exhaustion Signaling Pathway**	5.07	0.08	-0.277	ACVR2A,ACVR2B,CD274,FOS,GZMB,IL12A,IL6,IL6R,JUN,PDCD1LG2,SMAD3,STAT1,TGFBR2,TGFBR3
**Cell Cycle: G1/S Checkpoint Regulation**	7.21	0.164	-0.302	CCND1,CCND2,CDK2,CDKN1A,CDKN2A,CDKN2B,CDKN2D,E2F1,NRG1,SMAD3,TP53
**Corticotropin Releasing Hormone Signaling**	5.29	0.0897	-0.302	CACNA1C,CACNA1G,FOS,GLI1,ITPR1,JUN,NPR1,NPR3,NR4A1,POMC,PRKAR2B,PRKCE,SHH
**ERK/MAPK Signaling**	2.85	0.057	-0.302	ELF3,FOS,ITGA2,ITGA4,PLA2 G6,PRKAR2B,PRKCE,RAPGEF3,RAPGEF4,SRC,STAT1
**Signaling by Rho Family GTPases**	2.08	0.0451	-0.302	ACTA2,ACTB,CIT,FOS,ITGA2,ITGA4,JUN,NOX4,RND3,SLC9A1,VIM
**TGF-β Signaling**	6.47	0.125	-0.333	ACVR2A,ACVR2B,BCL2,BMP2,BMP4,BMPR1B,FOS,IRF7,JUN,SMAD3,SMAD9,TGFBR2
**Glioma Invasiveness Signaling**	4.9	0.122	-0.333	CD44,ITGB3,MMP2,MMP9,PLAU,RND3,TIMP1,TIMP4,VTN
**Gαs Signaling**	3.63	0.0841	-0.333	ADORA2A,GPER1,PRKAR2B,PTGER2,PTGER4,RAPGEF3,RAPGEF4,RGS2,SRC
**Cholecystokinin/Gastrin-mediated Signaling**	3.28	0.0756	-0.333	FOS,IL1RN,IL33,IL37,ITPR1,JUN,PRKCE,RND3,SRC
**GP6 Signaling Pathway**	3.28	0.0756	-0.333	COL12A1,COL2A1,COL3A1,COL8A1,ITGB3,ITPR1,LAMA3,LAMA5,PRKCE
**Protein Kinase A Signaling**	0.607	0.0251	-0.333	AKAP12,ITPR1,NGFR,PDE4D,PDE5A,PRKAR2B,PRKCE,SHH,SMAD3,TGFBR2
**IL-7 Signaling Pathway**	3.11	0.0897	-0.378	BAX,BCL2,CCND1,CDK2,IL7R,JUN,STAT1

**Calcium Signaling**	2.63	0.0534	-0.378	ACTA2,ATP2A3,ATP2B1,CACNA1C,CACNA1G,CALR,GRIN1,ITPR1,PRKAR2B,RCAN1,TRPC1
**VEGF Signaling**	2.5	0.0707	-0.378	ACTA2,ACTB,BCL2,FLT1,HIF1A,SRC,VEGFC
**Sumoylation Pathway**	2.41	0.068	-0.378	CEBPA,FAS,FOS,JUN,MYB,RND3,TP53
**GNRH Signaling**	2.17	0.052	-0.378	CACNA1C,CACNA1G,FOS,ITPR1,JUN,MMP2,PRKAR2B,PRKCE,SRC
**RhoA Signaling**	2	0.0569	-0.378	ACTA2,ACTB,CIT,IGF1,LPAR2,PFN1,RND3
**Small Cell Lung Cancer Signaling**	5.04	0.127	-0.447	BCL2,CCND1,CDK2,CDKN2B,E2F1,FHIT,IKBKE,RARB,TP53
**Bupropion Degradation**	3.99	0.2	-0.447	CYP1A1,CYP1B1,CYP2D6,CYP2E1,CYP3A5
**Acetone Degradation I (to Methylglyoxal)**	3.53	0.161	-0.447	CYP1A1,CYP1B1,CYP2D6,CYP2E1,CYP3A5
**Neurotrophin/TRK Signaling**	3.17	0.0921	-0.447	FOS,GAB1,JUN,NGF,NGFR,NTRK2,NTRK3
**Paxillin Signaling**	2.92	0.0741	-0.447	ACTA2,ACTB,ITGA2,ITGA4,ITGB3,ITGB4,ITGB8,SRC
**UVC-Induced MAPK Signaling**	2.52	0.098	-0.447	FOS,JUN,PRKCE,SRC,TP53
**Estrogen-Dependent Breast Cancer Signaling**	2.51	0.0811	-0.447	CCND1,FOS,HSD17B4,IGF1,JUN,SRC
**GDNF Family Ligand-Receptor Interactions**	2.45	0.0789	-0.447	FOS,GAB1,GDNF,IRS1,ITPR1,JUN
**Unfolded protein response**	2.35	0.0893	-0.447	BCL2,CALR,CEBPA,HSPA5,SREBF2
**Nicotine Degradation III**	2.31	0.0877	-0.447	CYP1A1,CYP1B1,CYP2D6,CYP2E1,CYP3A5
**Melatonin Degradation I**	2.22	0.0833	-0.447	CYP1A1,CYP1B1,CYP2D6,CYP2E1,CYP3A5
**Nicotine Degradation II**	2.07	0.0769	-0.447	CYP1A1,CYP1B1,CYP2D6,CYP2E1,CYP3A5
**Superpathway of Melatonin Degradation**	2.07	0.0769	-0.447	CYP1A1,CYP1B1,CYP2D6,CYP2E1,CYP3A5
**Chemokine Signaling**	1.71	0.0625	-0.447	CCL2,CXCL12,FOS,JUN,SRC
**NF-κB Activation by Viruses**	1.67	0.061	-0.447	IKBKE,ITGA2,ITGA4,ITGB3,PRKCE
**P2Y Purigenic Receptor Signaling Pathway**	1.43	0.0472	-0.447	FOS,ITGB3,JUN,P2RY6,PRKAR2B,PRKCE
**Cardiac β-adrenergic Signaling**	1.25	0.0426	-0.447	AKAP12,ATP2A3,CACNA1C,PDE4D,PDE5A,PRKAR2B

**ILK Signaling**	8.23	0.1	-0.5	ACTA2,ACTB,BMP2,CCND1,FOS,HIF1A,IRS1,ITGB3,ITGB4,ITGB8,JUN,MMP9,MUC1,RND3,SNAI1,SNAI2,TGFB1I1,VEGFC,VIM
**Sirtuin Signaling Pathway**	4.23	0.0584	-0.535	AGTRAP,BAX,CLOCK,CPS1,CPT1 A,E2F1,GADD45A,HIF1A,JUN,NDRG1,NR1 H2,PPARA,PPARGC1A,RARB,SDHC,SOD2,TP53
**Osteoarthritis Pathway**	21.9	0.166	-0.557	ACVRL1,ADAMTS5,AGER,ALPL,BGLAP,BMP2,CASP1,COL2A1,DCN,ELF3,FADD,FGF2,FGF8,FRZB,GLI1,HIF1A,IL1R1,IL1RL1,ITGA2,ITGA4,JAG1,LEP,MMP3,MMP9,PPARGC1A,PTHLH,S100A8,SMAD3,SMAD9,SOX9,SPP1,TGFBR2,TLR4,TNFRSF1B,VEGFC
**Wnt/β-catenin Signaling**	5.13	0.0809	-0.577	ACVR2A,ACVR2B,CCND1,CD44,CDKN2A,FRZB,JUN,POU5F1,RARB,SOX9,SRC,TGFBR2,TGFBR3,TP53
**Integrin Signaling**	3.56	0.061	-0.577	ACTA2,ACTB,CAV1,GSN,ITGA2,ITGA4,ITGB3,ITGB4,ITGB8,PDGFB,PFN1,RND3,SRC
**Insulin Secretion Signaling Pathway**	3.02	0.0535	-0.577	ABCC9,CACNA1C,CACNA1G,GCK,ITPR1,PRKAR2B,PRKCE,RAPGEF4,SCNN1B,SCNN1G,SLC2A4,SRC,STAT1
**Systemic Lupus Erythematosus In T Cell Signaling Pathway**	1.52	0.0359	-0.577	CASP1,CD44,FAS,FOS,GADD45A,IL6,ITPR1,JUN,LEP,ORAI1,RND3,STIM1
**White Adipose Tissue Browning Pathway**	3.68	0.0775	-0.632	ANGPT2,CACNA1C,CACNA1G,LEP,NPR1,PPARA,PPARGC1A,PRKAR2B,PRKG1,RARB
**Semaphorin Neuronal Repulsive Signaling Pathway**	3.65	0.0769	-0.632	CD44,ITGA2,ITGA4,NRP1,PDE4D,PRKAR2B,PRKG1,SEMA3A,TP53,VCAN
**Huntington's Disease Signaling**	3.64	0.0591	-0.632	BAX,CASP1,CTSD,EGF,HSPA5,IGF1,ITPR1,JUN,NGF,PENK,PRKCE,STX1A,TGM2,TP53
**Systemic Lupus Erythematosus In B Cell Signaling Pathway**	6.97	0.0764	-0.655	BCL2,CCND1,CCND2,FOS,GAB1,IL11,IL12A,IL15,IL33,IL37,IL6,IL6R,IL6ST,IRF7,JUN,LEP,LIF,OSM,PRKCE,SRC,STAT1
**ERK5 Signaling**	4.13	0.111	-0.707	CTF1,EGF,FOS,GAB1,IL6ST,LIF,NGF,SRC

**Agrin Interactions at Neuromuscular Junction**	3.88	0.103	-0.707	ACTA2,ACTB,ITGA2,ITGA4,ITGB3,JUN,NRG1, SRC
**Phospholipase C Signaling**	1.92	0.0428	-0.707	HMOX1,ITGA2,ITGA4,ITPR1,PLA2G6,PLD2,PR KCE,RAPGEF3,RND3,SRC,TGM2
**Thrombin Signaling**	1.3	0.0385	-0.707	EGF,F2RL2,GATA3,GATA4,ITPR1,PRKC E,RND3 ,SRC
**HOTAIR Regulatory Pathway**	6.26	0.0938	-0.775	CD44,CDKN1A,ESR2,EZH2,IRF1,MMP14,MMP1 5,MMP2,MMP28,MMP3,MMP9,SNAI2,SPP1,TL R4,VIM
**MIF Regulation of Innate Immunity**	3.83	0.143	-0.816	CD14,FOS,JUN,PLA2G6,TLR4,TP53
**PD-1, PD-L1 cancer immunotherapy pathway**	2.97	0.0755	-0.816	CD274,CDK2,IL12A,NGFR,PDCD1LG2,SMAD3, TNFRSF11B,TNFRSF1B
**Renin-Angiotensin Signaling**	2.67	0.0678	-0.816	CCL2,FOS,ITPR1,JUN,PRKAR2B,PRKCE,PTGE R2,STAT1
**JAK/Stat Signaling**	2.34	0.075	-0.816	CDKN1A,CISH,FOS,IL6,JUN,STAT1
**Relaxin Signaling**	2.05	0.0533	-0.816	FOS,JUN,MMP9,NPR1,NPR3,PDE4D,PDE5A,PR KAR2B
**Dopamine-DARPP32 Feedback in cAMP Signaling**	1.84	0.0491	-0.816	ATP2A3,CACNA1C,GRIN1,ITPR1,KCNJ2,PRKA R2B,PRKCE,PRKG1
**PI3K Signaling in B Lymphocytes**	1.74	0.0507	-0.816	C3,FOS,IKBKE,IRS1,ITPR1,JUN,TLR4
**Xenobiotic Metabolism General Signaling Pathway**	1.67	0.049	-0.816	AHR,FTL,HMOX1,NR1H2,NR1I2,PPARA,PRKC E
**CXCR4 Signaling**	1.35	0.0419	-0.816	CXCL12,FOS,ITPR1,JUN,PRKCE,RND3,SRC
**Regulation Of The Epithelial Mesenchymal Transition By Growth Factors Pathway**	12.5	0.128	-0.894	EGF,FGF1,FGF2,FGF7,FGF8,FOS,GAB1,IKBKE, IL6,IL6R,JUN,MMP2,MMP9,NGFR,PDGFA,PDG FB,PDGFRB,SMAD3,SNAI1,SNAI2,TGFBR2,TN FRSF11B,TNFRSF1B,VIM
**p53 Signaling**	9.18	0.153	-0.905	BAX,BCL2,CCND1,CCND2,CDK2,CDKN1A,CD KN2A,E2F1,FAS,GADD45A,HIF1 A,JUN,SERPIN E2,SNAI2,TP53
**Nitric Oxide Signaling in the Cardiovascular System**	5.46	0.111	-0.905	ATP2A3,BDKRB2,CACNA1C,CAV1,FLT1,ITPR1, PDE5A,PRKAR2B,PRKCE,PRKG1,VEGFC
**Ovarian Cancer Signaling**	6.26	0.101	-1	BCL2,CCND1,CD44,CDKN2A,E2F1,EDN1,EDN RA,EGF,MMP2,MMP9,PRKAR2B,SRC,TP53,VE GFC
**BMP signaling pathway**	4.4	0.106	-1	BMP2,BMP4,BMP6,BMPR1B,FST,JUN,NOG,PR KAR2B,SMAD9

**Th17 Activation Pathway**	4.17	0.0989	-1	AHR,HIF1A,IL12A,IL1R1,IL6,IL6R,PTGER2,PTGER4,RORA
**Inhibition of Angiogenesis by TSP1**	3.33	0.147	-1	JUN,MMP9,SDC1,TGFBR2,TP53
**Apelin Cardiac Fibroblast Signaling Pathway**	3.04	0.174	-1	ACE2,ANGPT2,APLNR,IL6
**TNFR2 Signaling**	2.6	0.133	-1	FOS,IKBKE,JUN,TNFRSF1B
**Cytotoxic T Lymphocyte-mediated Apoptosis of Target Cells**	2.39	0.118	-1	BCL2,FADD,FAS,GZMB
**Coagulation System**	2.35	0.114	-1	BDKRB2,PLAU,SERPINA5,TFPI
**Antiproliferative Role of TOB in T Cell Signaling**	2.18	0.103	-1	CDK2,SMAD3,TGFBR2,TOB1
**TNFR1 Signaling**	1.8	0.08	-1	FADD,FOS,IKBKE,JUN
**UVB-Induced MAPK Signaling**	1.74	0.0769	-1	FOS,JUN,PRKCE,TP53
**Thrombopoietin Signaling**	1.47	0.0635	-1	FOS,JUN,PRKCE,STAT1
**Netrin Signaling**	1.43	0.0615	-1	CACNA1C,CACNA1G,PRKAR2B,PRKG1
**IL-3 Signaling**	1.17	0.0506	-1	FOS,JUN,PRKCE,STAT1
**IL-8 Signaling**	11.1	0.115	-1.043	ANGPT1,ANGPT2,BAX,BCL2,CCND1,CCND2,CXCL1,EGF,FLT1,FOS,HMOX1,IKBKE,ITGB3,JUN,MMP2,MMP9,NOX4,PLD2,PRKCE,RND3,SRC,VCAM1,VEGFC
**Glioma Signaling**	7.55	0.127	-1.134	CCND1,CDKN1A,CDKN2 A,CDKN2B,CDKN2 D,E2F1,EGF,IGF1,IGF2R,PDGFA,PDGFB,PDGFRB,PRKCE,TP53
**PI3K/AKT Signaling**	6.49	0.0914	-1.134	BCL2,CCND1,CDKN1A,GAB1,IKBKE,IL13RA2,IL15RA,IL17RD,IL1R1,IL1RL1,IL6R,IL6ST,IL7R,ITGA2,ITGA4,TP53
**Actin Cytoskeleton Signaling**	5.23	0.0734	-1.134	ACTA2,ACTB,ARHGAP24,CD14,EGF,FGF1,FGF2,FGF7,FGF8,GSN,ITGA2,ITGA4,PDGFA,PDGFB,PFN1,SLC9A1
**Estrogen Biosynthesis**	4.91	0.171	-1.134	AKR1C3,CYP1A1,CYP1B1,CYP2D6,CYP2E1,CYP3A5,HSD17B4
**Neuregulin Signaling**	4.77	0.104	-1.134	BTC,DCN,EGF,ERBIN,ITGA2,ITGA4,NRG1,PRKCE,RPS6,SRC
**PEDF Signaling**	2.97	0.0854	-1.134	BCL2,FAS,GDNF,IKBKE,NGF,SOD2,TP53
**Ceramide Signaling**	2.8	0.0795	-1.134	BCL2,CTSD,FOS,JUN,NGFR,TNFRSF11B,TNFRSF1B

**Fcγ Receptor-mediated Phagocytosis in Macrophages and Monocytes**	2.63	0.0745	-1.134	ACTA2,ACTB,HMOX1,PLA2G6,PLD2,PRKCE,SRC
**mTOR Signaling**	1.67	0.0429	-1.134	HIF1A,HMOX1,IRS1,PLD2,PRKCE,RND3,RPS23,RPS6,VEGFC
**AMPK Signaling**	4.7	0.0701	-1.155	ACTB,CCND1,CDKN1A,CFTR,CPT1A,EEF2,FASN,HMGCR,IRS1,LEP,ORAI1,PPARGC1 A,PRKAR2B,SLC2A4,STIM1
**LPS/IL-1 Mediated Inhibition of RXR Function**	10.9	0.107	-1.213	ABCB1,ABCC2,ALDH1A2,APOC1,APOE,CD14,CPT1A,CYP3A5,IL1R1,IL1RL1,IL1RN,IL33,IL37,JUN,NDST2,NGFR,NR1H2,NR1I2,PPARA,PPARGC1A,SULT1E1,TLR4,TNFRSF11B,TNFRSF1B
**Androgen Signaling**	4.15	0.0809	-1.265	CACNA1C,CACNA1G,CALR,CCND1,JUN,PRKAR2B,PRKCE,SHBG,SMAD3,SRC,TGFB1I1
**Synaptic Long Term Depression**	2.41	0.0529	-1.265	CACNA1C,CACNA1G,IGF1,ITPR1,NPR1,NPR3,PLA2G6,PNPLA3,PRKCE,PRKG1
**Factors Promoting Cardiogenesis in Vertebrates**	6.63	0.1	-1.291	ACVR2A,ACVR2B,BMP2,BMP4,BMP6,BMPR1B,CCND1,GATA4,MYOCD,NOG,NOX4,PR KCE,SMAD9,TGFBR2,TGFBR3
**HIF1α Signaling**	16.2	0.141	-1.3	BMP6,CDKN1A,EDN1,EGF,FGF2,FLT1,GCK,HIF1A,HMOX1,HSPA5,IGF1,IL6,IL6R,JUN,MMP14,MMP15,MMP2,MMP28,MMP3,MMP9,NOX4,PDGFB,PKM,PRKCE,RPS6,SLC2A4,TP53,VEGFC,VIM
**Bladder Cancer Signaling**	12.4	0.186	-1.342	CCND1,CDKN1A,CDKN2 A,E2F1,EGF,FGF1,FGF2,FGF7,FGF8,MMP14,MMP15,MMP2,MMP28,MMP3,MMP9,RASSF1,TP53,VEGFC
**Growth Hormone Signaling**	3.35	0.0986	-1.342	CEBPA,FOS,IGF1,IRS1,PRKCE,SLC2A4,STAT1
**Retinoate Biosynthesis I**	3.33	0.147	-1.342	AKR1C3,ALDH1A2,BMP2,DHRS3,RDH5
**IGF-1 Signaling**	2.39	0.0673	-1.342	CCN3,FOS,IGF1,IGFBP2,IRS1,JUN,PRKAR2B

**Role of CHK Proteins in Cell Cycle Checkpoint Control**	2.31	0.0877	-1.342	CDK1,CDK2,CDKN1A,E2F1,TP53
**CDK5 Signaling**	1.73	0.0556	-1.342	ITGA2,LAMA5,NGF,NGFR,NTRK2,PRKAR2B
**Apelin Adipocyte Signaling Pathway**	1.67	0.061	-1.342	APLNR,HIF1A,NOX4,PPARGC1A,PRKAR2B
**IL-1 Signaling**	1.5	0.0549	-1.342	FOS,IKBKE,IL1R1,JUN,PRKAR2B
**Melanocyte Development and Pigmentation Signaling**	1.44	0.0532	-1.342	BCL2,KITLG,POMC,PRKAR2B,SRC
**Crosstalk between Dendritic Cells and Natural Killer Cells**	5.06	0.112	-1.414	ACTA2,ACTB,FAS,IL12A,IL15,IL15RA,IL6,MICA,TLR4,TNFRSF1B
**Gαq Signaling**	1.92	0.0506	-1.414	HMOX1,HTR2A,IKBKE,ITPR1,PLD2,PRKCE,RGS2,RND3
**Leukocyte Extravasation Signaling**	12.1	0.122	-1.46	ACTA2,ACTB,CD44,CLDN4,CXCL12,CYBA,ITGA2,ITGA4,ITGB3,MMP14,MMP15,MMP2,MMP28,MMP3,MMP9,PECAM1,PRKCE,RAPGEF3,RAPGEF4,SRC,THY1,TIMP1,TIMP4,VCAM1
**Role of PKR in Interferon Induction and Antiviral Response**	11.9	0.162	-1.5	BAX,CASP1,E2F1,FADD,FAS,FOS,HSPA5,IKBKE,IRF1,JUN,MSR1,NPM1,PDGFA,PDGFB,PDGFRB,SCARA5,STAT1,TLR4,TP53
**IL-15 Production**	4.62	0.0909	-1.508	DDR1,FLT1,IL15,IL6,IRF1,MERTK,NTRK2,NTRK3,PDGFRB,SRC,STAT1
**Acute Phase Response Signaling**	11.2	0.123	-1.606	C2,C3,FOS,FTL,HMOX1,HP,IKBKE,IL1R1,IL1RN,IL33,IL37,IL6,IL6R,IL6ST,JUN,NGFR,OSM,SAA1,SERPINA3,SOD2,TNFRSF11B,TNFRSF1B
**VDR/RXR Activation**	6.52	0.141	-1.633	BGLAP,CD14,CDKN1A,CEBPA,GADD45A,IL12A,IL1RL1,KLF4,PDGFA,PRKCE,SPP1
**Role of Pattern Recognition Receptors in Recognition of Bacteria and Viruses**	5.72	0.0909	-1.633	C3,CASP1,IL11,IL12A,IL15,IL33,IL37,IL6,IRF7,LEP,LIF,OSM,PRKCE,TLR4
**iNOS Signaling**	4.63	0.156	-1.633	CD14,FOS,IKBKE,IRF1,JUN,STAT1,TLR4
**Oncostatin M Signaling**	3.77	0.14	-1.633	CHI3L1,IL6ST,MMP3,OSM,PLAU,STAT1
**Activation of IRF by Cytosolic Pattern Recognition Receptors**	2.87	0.0952	-1.633	FADD,IKBKE,IL6,IRF7,JUN,STAT1

**Regulation of Actin-based Motility by Rho**	2.63	0.0745	-1.633	ACTA2,ACTB,GSN,ITGA2,ITGA4,PFN1,RND3
**Prolactin Signaling**	2.31	0.0741	-1.633	FOS,IRF1,IRS1,JUN,PRKCE,STAT1
**LPS-stimulated MAPK Signaling**	2.29	0.0732	-1.633	CD14,FOS,IKBKE,JUN,PRKCE,TLR4
**Regulation Of The Epithelial Mesenchymal Transition In Development Pathway**	2.24	0.0714	-1.633	GLI1,HIF1A,JAG1,SHH,SNAI1,SNAI2
**ErbB Signaling**	2	0.0638	-1.633	BTC,EGF,FOS,JUN,NRG1,PRKCE
**GPCR-Mediated Nutrient Sensing in Enteroendocrine Cells**	1.66	0.0536	-1.633	CACNA1C,CACNA1G,ITPR1,PRKAR2B,PRKCE ,RAPGEF4
**NGF Signaling**	1.63	0.0526	-1.633	BAX,GAB1,IKBKE,NGF,NGFR,TP53
**G Beta Gamma Signaling**	1.5	0.0492	-1.633	CACNA1C,CACNA1G,CAV1,PRKAR2B,PR KCE, SRC
**Sperm Motility**	6.41	0.0807	-1.667	CACNA1G,DDR1,FLT1,ITPR1,MERTK,NPPC,NP R1,NTRK2,NTRK3,PDE4D,PDGFRB,PLA2G6,P NPLA3,PRKAR2B,PRKCE,PRKG1,SLC12A2,SR C
**Apelin Endothelial Signaling Pathway**	4.09	0.087	-1.667	ANGPT1,APLNR,CCL2,FOS,HIF1A,JUN,KLF2,P RKCE,SMAD3,VCAM1
**Type II Diabetes Mellitus Signaling**	3.98	0.0775	-1.667	CACNA1C,CACNA1G,GCK,IKBKE,IRS1,NGFR, PKM,PRKCE,SLC2A4,TNFRSF11B,TNFRSF1B
**FAT10 Cancer Signaling Pathway**	10.3	0.261	-1.732	ACKR3,ACVR2A,ACVR2B,IKBKE,IL6,NGFR,S MAD3,TGFBR2,TGFBR3,TNFRSF11B,TNFRSF1 B,TP53
**Tec Kinase Signaling**	4.06	0.0732	-1.732	ACTA2,ACTB,FADD,FAS,FOS,ITGA2,ITGA4,PR KCE,RND3,SRC,STAT1,TLR4
**Xenobiotic Metabolism PXR Signaling Pathway**	3.42	0.0625	-1.732	ABCB1,ABCC2,ALDH1A2,CDK2,CES1,CYP3A5 ,NDST2,NR1I2,PPARGC1A,PRKAR2B,PRKCE,S ULT1E1
**Colorectal Cancer Metastasis Signaling**	12.1	0.107	-1.8	BAX,CCND1,EGF,FOS,IL6,IL6R,IL6ST,JUN,MM P14,MMP15,MMP2,MMP28,MMP3,MMP9,PRKA R2B,PTGER1,PTGER2,PTGER3,PTGER4,RND3, SMAD3,SRC,STAT1,TGFBR2,TLR4,TP53,VEGF C
**HMGB1 Signaling**	10.1	0.121	-1.807	AGER,CCL2,FOS,IL11,IL12A,IL15,IL1R1,IL33,I L37,IL6,JUN,LEP,LIF,NGFR,OSM,RND3,TLR4,T NFRSF11B,TNFRSF1B,VCAM1

**Toll-like Receptor Signaling**	5.69	0.132	-1.89	CD14,FOS,IL12A,IL1RL1,IL1RN,IL33,IL37,JUN, PPARA,TLR4
**Death Receptor Signaling**	2.71	0.0769	-1.89	ACTA2,ACTB,BCL2,FADD,FAS,IKBKE,TNFRSF 1B
**NRF2-mediated Oxidative Stress Response**	2.41	0.0529	-1.89	ABCC2,ACTA2,ACTB,FOS,FTH1,FTL,HMOX1,J UN,PRKCE,SOD2
**BEX2 Signaling Pathway**	5.54	0.127	-1.897	BCL2,CCND1,CDKN1A,HIF1A,JUN,MMP2,NGF, NGFR,SPP1,VEGFC
**Xenobiotic Metabolism CAR Signaling Pathway**	2.41	0.0529	-1.897	ABCB1,ABCC2,ALDH1A2,CYP1A1,CYP3 A5,N DST2,PPARGC1A,PRKCE,SRC,SULT1E1
**Pancreatic Adenocarcinoma Signaling**	10.5	0.156	-1.941	BCL2,CCND1,CDK2,CDKN1A,CDKN2 A,CDKN 2B,CYP2E1,E2F1,EGF,HMOX1,MMP9,PLD2,SM AD3,STAT1,TGFBR2,TP53,VEGFC
**Tumoricidal Function of Hepatic Natural Killer Cells**	2.97	0.167	-2	BAX,FADD,FAS,GZMB
**Role of BRCA1 in DNA Damage Response**	2.34	0.075	-2	ACTB,CDKN1A,E2F1,GADD45A,STAT1,TP53
**BAG2 Signaling Pathway**	2.03	0.093	-2	CDKN1A,CTSB,HSPA5,TP53
**Coronavirus Pathogenesis Pathway**	9.99	0.127	-2.065	ACE2,BAX,BCL2,CASP1,CCL2,CCND1,CDK2,E 2F1,FOS,IL6,IRF7,JUN,NPM1,RPS23,RPS6,SMA D3,STAT1,TGFBR2,TP53
**Production of Nitric Oxide and Reactive Oxygen Species in Macrophages**	8.31	0.101	-2.065	APOA4,APOC1,APOD,APOE,CLU,CYBA,FOS,I KBKE,IRF1,JUN,NGFR,PPARA,PRKCE,RND3,S 100A8,STAT1,TLR4,TNFRSF11B,TNFRSF1B
**Myc Mediated Apoptosis Signaling**	8.62	0.22	-2.111	BAX,BCL2,CDKN2A,FADD,FAS,IKBKE,NGFR, PRKAR2B,TNFRSF11B,TNFRSF1B,TP53
**Cell Cycle: G2/M DNA Damage Checkpoint Regulation**	2.6	0.102	-2.236	CDK1,CDKN1A,CDKN2 A,GADD45A,TP53
**ATM Signaling**	1.94	0.0619	-2.236	CDK1,CDK2,CDKN1A,GADD45A,JUN,TP53
**Xenobiotic Metabolism AHR Signaling Pathway**	1.61	0.0588	-2.236	AHR,ALDH1A2,CYP1A1,CYP1B1,IL6

**Estrogen Receptor Signaling**	10.9	0.0884	-2.268	BCL2,CACNA1C,CAV1,CCND1,CDKN1A,EGF,ESR2,FOS,GPER1,HIF1A,IGF1,IGF2R,JUN,LEP,MMP14,MMP15,MMP2,MMP28,MMP3,MMP9,PPARGC1A,PRKAR2B,PRKCE,SDHC,SNAI1,SOD2,SRC,TP53,VEGFC
**Role of NFAT in Cardiac Hypertrophy**	4.7	0.0701	-2.324	CACNA1C,CACNA1G,CTF1,GATA4,IGF1,IL11,IL6,IL6ST,ITPR1,LIF,PRKAR2B,PRKCE,RCAN1,SRC,TGFBR2
**Induction of Apoptosis by HIV1**	5.6	0.148	-2.333	BAX,BCL2,FADD,FAS,IKBKE,NGFR,TNFRSF11B,TNFRSF1B,TP53
**Neuroinflammation Signaling Pathway**	14.3	0.107	-2.335	ACVR2A,ACVR2B,AGER,BCL2,CASP1,CCL2,CX3CL1,CXCL12,FAS,FOS,GABRB2,GDNF,GRIN1,HMOX1,IKBKE,IL12A,IL1R1,IL6,IL6R,IRF7,JUN,MMP3,MMP9,NGF,NOX4,PLA2G6,SOD2,STAT1,TGFBR2,TGFBR3,TLR4,VCAM1
**STAT3 Pathway**	19.2	0.2	-2.357	BCL2,BMP6,BMPR1B,CDKN1A,CISH,EGF,FGF2,FLT1,IGF1,IGF2R,IL13RA2,IL15RA,IL17RD,IL1R1,IL1RL1,IL6R,IL6ST,IL7R,NGFR,NTRK2,NTRK3,PDGFB,PDGFRB,SRC,TGFBR2,TGFBR3,TNFRSF11A
**TREM1 Signaling**	2.48	0.08	-2.449	CASP1,CCL2,CXCL3,IL1RL1,IL6,TLR4
**Inhibition of ARE-Mediated mRNA Degradation Pathway**	1.5	0.0492	-2.449	NGFR,PABPN1,POMC,PRKAR2B,TNFRSF11B,TNFRSF1B
**IL-6 Signaling**	9.49	0.136	-2.5	ABCB1,CD14,FOS,IKBKE,IL1R1,IL1RL1,IL1RN,IL33,IL37,IL6,IL6R,IL6ST,JUN,NGFR,TNFAIP6,TNFRSF11B,TNFRSF1B
**Dendritic Cell Maturation**	6.23	0.0874	-2.5	CD1D,COL2A1,COL3A1,IKBKE,IL12A,IL15,IL1RN,IL33,IL37,IL6,LEP,NGFR,STAT1,TLR4,TNFRSF11B,TNFRSF1B
**Thyroid Cancer Signaling**	5.54	0.127	-2.53	CCND1,FOS,GDNF,IGF1,IRS1,JUN,NGF,NTRK2,NTRK3,TP53
**p38 MAPK Signaling**	5.5	0.102	-2.53	FADD,FAS,IL1R1,IL1RL1,IL1RN,IL33,IL37,PLA2G6,STAT1,TGFBR2,TNFRSF1B,TP53
**Cardiac Hypertrophy Signaling**	1.72	0.0417	-2.53	CACNA1C,GATA4,IGF1,IL6,IL6R,IRS1,JUN,PRKAR2B,RND3,TGFBR2

**Senescence Pathway**	9.75	0.0909	-2.6	ACVR2A,ACVR2B,CACNA1C,CCND1,CDK1 ,CDK2,CDKN1A,CDKN2A,CDKN2B,E2F1,ELF3,EZH2,GADD45A,GATA4,IKB KE,IL6,JUN,NF1,SAA1,SMAD3,SMAD9,SOD2,TGFBR2,TGFBR3,TP53
**Apelin Liver Signaling Pathway**	6.33	0.269	-2.646	APLNR,COL2A1,COL3A1,EDN1,FAS,IRS1,PDGFRB
**Type I Diabetes Mellitus Signaling**	5.78	0.108	-2.714	BCL2,FADD,FAS,GZMB,IKBKE,IL12A,IL1R1,IRF1,NGFR,STAT1,TNFRSF11B,TNFR SF1B
**Necroptosis Signaling Pathway**	3.6	0.0701	-2.714	CASP1,FADD,FAS,MERTK,NGFR,PLA2 G6,STAT1,TLR4,TNFRSF11B,TNFRSF1B,TP53
**Hepatic Fibrosis Signaling Pathway**	28.9	0.141	-2.774	ACTA2,ACVR2A,ACVR2B,BCL2,CACNA1C,CCL2,CCND1,COL2A1,COL3A1,EDN1,EDNRA,EZH2,FGF2,FLT1,FOS,FTH1,FTL,GLI1,HIF1 A,IKBKE,IL1R1,IL1RL1,IL1RN,IL33,IL37,ITGA2,ITGA4,ITGB3,JUN,LEP,NGFR,NOX4,PDGFA,PDGFB,PDGFRB,PRKAR2B,PRKCE,RND3,SHH,SMAD3,SNAI1,SOD2,SPP1,TFRC,TGFBR2,TGFBR3,TIMP1,TLR4,TNFRSF11B,TNFRSF1B,VCAM1,VEGFC
**Cardiac Hypertrophy Signaling (Enhanced)**	22.2	0.105	-3.355	ACVR2A,ACVR2B,ATP2A3,CACNA1C,CTF1,EDN1,EDNRA,EDNRB,FGF1,FGF2,FGF7,FGF8,GATA4,IGF1,IKBKE,IL11,IL12A,IL13RA2,IL15,IL15RA,IL17RD,IL1R1,IL1RL1,IL33,IL37,IL6,IL6R,IL6ST,IL7R,ITGA2,ITGA4,ITPR1,JUN,LEP,LIF,MYOCD,NGFR,NPR1,OSM,PDE4 D,PDE5A,PRKAR2B,PRKCE,PRKG1,RCAN1,RPS6,TG,TGFBR2,TGFBR3,TNFRSF11B,TNFRSF1B
**NF-κB Signaling**	11.2	0.123	-3.411	BMP2,BMP4,BMPR1B,EGF,FADD,FLT1,IGF2R,IL1R1,IL1RN,IL33,IL37,NGF,NGFR,NTRK2,NTRK3,PDGFRB,TGFBR2,TGFBR3,TLR4,TNFRSF11A,TNFRSF11B,TNFRSF1B

Supplemental Table S2 – 10 Most Significant Networks from IPA

Network ID	Molecules in Network	Score	Focus Molecules	Top Diseases and Functions
**1**	Alp,ALPL,BGLAP,BGN,BMP2,BMP4,BMPR1B,CDKN1A,CIT,COL12A1,COL2A1,COL8A1,CTSK,DDR1,DLL1,DLL4,FGF8,GLI1,Histone h2a,HSD17B4,IL37,Importin beta,Inflammasome (Nalp1, Asc, Casp1, Casp4),ITGB8,KLF4,Lysosomal Protease,MSX2,Notch,PTHLH,SHH,SOX9,TCF,Tgf beta,TGFB1I1,TIP60	41	26	[Connective Tissue Development and Function, Skeletal and Muscular System Development and Function, Tissue Development]
**2**	ACADVL,ADAMTS5,ADAMTS9,AGER,APOE,Cbp/p300,CD44,CLDN4,CSF1,CSPG,CTSB,CXCL12,CXCL3,CYP1A1,CYP2D6,CYP2E1,EGFR ligand,estrogen receptor,IL1,LPAM,Metalloprotease,Mitochondrial complex 1,MMP14,MMP2,MMP9,PLAU,RGS2,SALL4,SPP1,SPTLC2,Tnf (family),TNFRSF11A,TPT1,VCAM1,VCAN	41	26	[Cancer, Organismal Injury and Abnormalities, Tumor Morphology]
**3**	AIM2 Inflammasome,CASP1,CD14,CISH,CSF3,CXCL2,DPP4,Gα12/13,hemoglobin,HMOX1,Hsp70,Ifn gamma,Iga,Ige,IGF1,IL-2R,IL11,IL12 (complex),IL13RA2,IL15,IL6ST,IL7R,KITLG,LIF,MYB,NCAM1,OSM,PABPN1,PAPPA,STAT5a/b,TFRC,TLR4,TNFR/Fas,TNFRSF1B,VEGFC	36	24	[Cell-To-Cell Signaling and Interaction, Cellular Development, Cellular Growth and Proliferation]
**4**	ACTA2,Ap1,CEBPA,CG,CLOCK,CLU,CPS1,Creb,CRY1,FBLN1,FGF1,FGF2,FOSL2,FSH,GRN,GTPase,Histone h3,KRT8,LEP,Lh,MAP2K1/2,Mek,MKI67,NPPC,NR4A1,NRP1,PPARA,PROM2,Raf,Ras homolog,RASSF1,SMAD3,Syntaxin1,TGM2,TK1	34	23	[Cellular Movement, Organismal Injury and Abnormalities, Tissue Morphology]

**5**	ACTB,AHR,alcohol group acceptor phosphotransferase,APOD,BAX,CAV1,CD3,CDK2,CDKN2A,E2F1,FADD,GATA3,HIF1A,Hsp90,IKBKE,IKK (complex),IMPDH2,Interferon alpha,MX1,NPM1,NRG1,P-TEFb,p85 (pik3r),PRKCE,Rac,RNA polymerase II,Rnr,RPL31,RPS23,RPS6,SF3B1,SNAI1,SOD2,TCR,Ubiquitin	34	23	[Cancer, Cell Death and Survival, Organismal Injury and Abnormalities]
**6**	Alpha catenin,ANTXR1,CBS/CBSL,CDO1,Collagen type V,Collagen Type XI,Collagen(s),collagenase,CONNEXIN,CXCL1,CYBA,DNA-methyltransferase,ELN,FHIT,gelatinase,ITGB4,KCNMA1,LAMA5,Laminin (complex),Laminin1,MMP3,NDRG1,NES,Pka,Pka catalytic subunit,Ppp2c,PRC2,PTGES,Ral,Rap1,S100A8,SNAI2,SPARC,TNFAIP6,VIM	26	19	[Cardiovascular System Development and Function, Cellular Movement, Organismal Development]
**7**	ABCC9,ACAC,ACE2,AGTRAP,Alpha actin,ATPase,C1QTNF5,CNN1,CST3,CTF1,endothelin receptor,FOS,Gata,GATA4,Hedgehog,KATP Channel,KCNE3,Kcnj,KCNJ2,L-type Calcium Channel,mediator,MEF2,MYOCD,Myosin,NPR1,PDPN,PRKG1,sGC,SLC9A3,SMOOTH MUSCLE ACTIN,Tni,TOR2A,Tropomyosin,Troponin t,UCN2	24	18	[Cardiovascular Disease, Cardiovascular System Development and Function, Organismal Development]

**8**	3 beta HSD,AKAP12,ANG,CAMK2,CCND1,CD3 group,CPT1A,creatine kinase,FAM20A,FASN,FHL1,GABP,GCH1 ,glutathione peroxidase,Growth hormone,Hdac,histone deacetylase,Histone h4,HPSE,HSD11B1,HSP,Ldh (complex),LDL-cholesterol,MUC1,NDST2,NF1,PFK,Pgk,PI3K (family),PKM,PPARGC1A,PRKAA,PRKAR2B,SULT1E1,TXNIP	24	18	[Gastrointestinal Disease, Hepatic System Disease, Liver Steatosis]
**9**	ACKR3,Adaptor protein 2,ADORA1,APLNR,BDKRB2,Beta Arrestin,Clathrin,cytokine,EDN1,EDNRA,EDNRB,Endothelin,F2RL2,G protein alpha,G protein alphai,Girk,GNRH,Gpcr,GPER1,HRH4,HTR2A,LPAR2,Nos,P glycoprotein,P110,Pik3r,PTGER1,PTGER2,PTGER3,PTGER4,receptor protein tyrosine kinase,Relaxin,SSTR1,SSTR3,voltage-gated calcium channel	24	18	[Cardiovascular System Development and Function, Cell-To-Cell Signaling and Interaction, Hematological System Development and Function]
**10**	ABCC11,ADCY8,ADRB,CACNA1 G,Calmodulin,CFTR,CRYAB,ERBIN,ESR2,EZH2,F2RL1,Focal adhesion kinase,FTH1,G protein beta gamma,GALR2,GPR26,HISTONE,Hsf4,HSPA5,ITGA4,LPAR2,NPBWR1,P2RY6,PABPC5,PLC,POMC,PON2,POU5F1,SDR16C5,SLC9A3R1,STAT,STX1A,SYNE1,TAAR1,tubulin	24	18	[Cell-To-Cell Signaling and Interaction, Cellular Function and Maintenance, Molecular Transport]

Supplemental Table S3 - Pathways Associated with Cardiovascular Signaling from IPA

Ingenuity Canonical Pathways	-log(p-value)	Ratio	z-score	Molecules
**Cardiac Hypertrophy Signaling (Enhanced)**	22.2	0.105	-3.355	ACVR2A,ACVR2B,ATP2A3,CACNA1C,CTF1,EDN1,EDNRA,EDNRB,FGF1,FGF2,FGF7,FGF8,GATA4,IGF1,IKBKE,IL11,IL12A,IL13RA2,IL15,IL15RA,IL17RD,IL1R1,IL1RL1,IL33,IL37,IL6,IL6R,IL6ST,IL7R,ITGA2,ITGA4,ITPR1,JUN,LEP,LIF,MYOCD,NGFR,NPR1,OSM,PDE4 D,PDE5A,PRKAR2B,PRKCE,PRKG1,RCAN1,RPS6,TG,TGFBR2,TGFBR3,TNFRSF11B,TNFRSF1B
**Atherosclerosis Signaling**	16.5	0.189	NA	APOA4,APOC1,APOD,APOE,CCL2,CLU,COL2 A1,COL3A1,CSF1,CXCL12,IL1RN,IL33,IL37,IL6,ITGA4,MMP3,MMP9,MSR1,PDGFA,PDGFB,PLA2 G6,PNPLA3,S100A8,VCAM1
**HIF1α Signaling**	16.2	0.141	-1.3	BMP6,CDKN1A,EDN1,EGF,FGF2,FLT1,GCK,HIF1A,HMOX1,HSPA5,IGF1,IL6,IL6R,JUN,MMP14,MMP15,MMP2,MMP28,MMP3,MMP9,NOX4,PDGFB,PKM,PRKCE,RPS6,SLC2A4,TP53,VEGFC,VIM
**Factors Promoting Cardiogenesis in Vertebrates**	6.63	0.1	-1.291	ACVR2A,ACVR2B,BMP2,BMP4,BMP6,BMPR1B,CCND1,GATA4,MYOCD,NOG,NOX4,PR KCE,SMAD9,TGFBR2,TGFBR3
**Nitric Oxide Signaling in the Cardiovascular System**	5.46	0.111	-0.905	ATP2A3,BDKRB2,CACNA1C,CAV1,FLT1,ITPR1,PDE5A,PRKAR2B,PRKCE,PRKG1,VEGFC
**Endothelin-1 Signaling**	5.38	0.0798	0.258	CASP1,EDN1,EDNRA,EDNRB,FOS,GAB1,HMOX1,ITPR1,JUN,PLA2G6,PLD2,PNPLA3,PRKCE,PTGER2,SRC
**Role of NFAT in Cardiac Hypertrophy**	4.7	0.0701	-2.324	CACNA1C,CACNA1G,CTF1,GATA4,IGF1,IL11,IL6,IL6ST,ITPR1,LIF,PRKAR2B,PRKCE,RCAN1,SRC,TGFBR2
**Adrenomedullin signaling pathway**	4.49	0.0711	0	BAX,BCL2,C3,FOS,HIF1A,IL1RN,IL33,IL37,ITPR1,MMP2,NPR1,NPR3,PRKAR2B,PRKG1
**Apelin Endothelial Signaling Pathway**	4.09	0.087	-1.667	ANGPT1,APLNR,CCL2,FOS,HIF1A,JUN,KLF2,PRKCE,SMAD3,VCAM1
**eNOS Signaling**	3.55	0.0692	0.632	BDKRB2,CAV1,ESR2,FLT1,HSPA5,ITPR1,LPAR2,PRKAR2B,PRKCE,PRKG1,VEGFC
**Inhibition of Angiogenesis by TSP1**	3.33	0.147	-1	JUN,MMP9,SDC1,TGFBR2,TP53
**Cardiomyocyte Differentiation via BMP Receptors**	3.28	0.2	NA	BMP2,BMP4,BMPR1B,GATA4

**Apelin Cardiac Fibroblast Signaling Pathway**	3.04	0.174	-1	ACE2,ANGPT2,APLNR,IL6
**Renin-Angiotensin Signaling**	2.67	0.0678	-0.816	CCL2,FOS,ITPR1,JUN,PRKAR2B,PRKCE,PTGER 2,STAT1
**Apelin Cardiomyocyte Signaling Pathway**	2.5	0.0707	0	APLNR,ATP2A3,HIF1A,ITPR1,PRKCE,SLC9A1,S LC9A3
**Cellular Effects of Sildenafil (Viagra)**	2.39	0.0611	NA	ACTA2,ACTB,CACNA1C,ITPR1,PDE4D,PDE5A, PRKAR2B,PRKG1
**Aldosterone Signaling in Epithelial Cells**	1.92	0.0506	0	CRYAB,HSPA5,ITPR1,PRKCE,SCNN1B,SC NN1 G, SLC12A2,SLC9A1
**Hypoxia Signaling in the Cardiovascular System**	1.84	0.0676	0	EDN1,HIF1A,JUN,SLC2A4,TP53
**Cardiac Hypertrophy Signaling**	1.72	0.0417	-2.53	CACNA1C,GATA4,IGF1,IL6,IL6R,IRS1,JUN,PRK AR2B,RND3,TGFBR2
**P2Y Purigenic Receptor Signaling Pathway**	1.43	0.0472	-0.447	FOS,ITGB3,JUN,P2RY6,PRKAR2B,PRKCE
**Thrombin Signaling**	1.3	0.0385	-0.707	EGF,F2RL2,GATA3,GATA4,ITPR1,PRKC E,RND3, SRC
**Cardiac β-adrenergic Signaling**	1.25	0.0426	-0.447	AKAP12,ATP2A3,CACNA1C,PDE4D,PDE5A,PRK AR2B

Supplemental Table S4 – DAVID and PANTHER GO Term Intersections based on all DEGs

Biological Process

Database	ID	GO Term	Count	Fold Enrichment	P-Value	FDR
**DAVID**	GO:0009653	Anatomical Structure Morphogenesis	31	2.1	1.10E-04	2.78E-02
**PANTHER**	GO:0009653	Anatomical Structure Morphogenesis	364	1.4	3.82E-11	4.23E-08
**DAVID**	GO:0001525	Angiogenesis	49	1.8	3.69E-05	1.24E-02
**PANTHER**	GO:0001525	Angiogenesis	69	1.8	2.11E-05	2.15E-03
**DAVID**	GO:0007411	Axon Guidance	46	2.3	1.01E-07	2.03E-04
**PANTHER**	GO:0007411	Axon Guidance	57	2.2	1.08E-06	1.82E-04
**DAVID**	GO:0031103	Axon Regeneration	9	5.1	1.41E-04	3.16E-02
**PANTHER**	GO:0031103	Axon Regeneration	11	3.9	6.06E-04	2.83E-02
**DAVID**	GO:0016339	Calcium-Dependent Cell-Cell Adhesion Via Plasma Membrane Cell Adhesion Molecules	18	3.6	3.40E-06	3.15E-03
**PANTHER**	GO:0016339	Calcium-Dependent Cell-Cell Adhesion Via Plasma Membrane Cell Adhesion Molecules	18	3.5	4.33E-05	3.89E-03
**DAVID**	GO:0060070	Canonical Wnt Signaling Pathway	26	2.4	3.59E-05	1.24E-02
**PANTHER**	GO:0060070	Canonical Wnt Signaling Pathway	26	2.2	7.70E-04	3.35E-02
**DAVID**	GO:0007155	Cell Adhesion	123	2.1	1.89E-15	1.15E-11
**PANTHER**	GO:0007155	Cell Adhesion	201	1.9	2.14E-14	3.68E-11
**DAVID**	GO:0051301	Cell Division	68	1.7	1.83E-05	6.94E-03
**PANTHER**	GO:0051301	Cell Division	92	1.6	1.33E-04	9.40E-03
**DAVID**	GO:0098609	Cell-Cell Adhesion	38	1.9	2.32E-04	4.01E-02
**PANTHER**	GO:0098609	Cell-Cell Adhesion	121	2.0	1.40E-10	1.28E-07
**DAVID**	GO:0007267	Cell-Cell Signaling	52	2.2	1.40E-07	2.12E-04
**PANTHER**	GO:0007267	Cell-Cell Signaling	185	1.5	3.78E-07	8.77E-05
**DAVID**	GO:0050965	Detection Of Temperature Stimulus Involved In Sensory Perception Of Pain	11	5.3	1.02E-05	4.43E-03
**PANTHER**	GO:0050965	Detection Of Temperature Stimulus Involved In Sensory Perception Of Pain	11	4.9	1.37E-04	9.61E-03
**DAVID**	GO:0030198	Extracellular Matrix Organization	37	1.9	1.86E-04	3.65E-02
**PANTHER**	GO:0030198	Extracellular Matrix Organization	53	1.7	6.16E-04	2.86E-02
**DAVID**	GO:0007156	Homophilic Cell Adhesion Via Plasma Membrane Adhesion Molecules	51	2.8	1.39E-11	4.22E-08

**PANTHER**	GO:0007156	Homophilic Cell Adhesion Via Plasma Membrane Adhesion Molecules	50	2.6	2.55E-08	7.93E-06
**DAVID**	GO:0000278	Mitotic Cell Cycle	32	2.0	1.70E-04	3.56E-02
**PANTHER**	GO:0000278	Mitotic Cell Cycle	117	1.7	5.49E-07	1.18E-04
**DAVID**	GO:0000070	Mitotic Sister Chromatid Segregation	13	3.4	2.45E-04	4.12E-02
**PANTHER**	GO:0000070	Mitotic Sister Chromatid Segregation	38	2.5	5.24E-06	6.95E-04
**DAVID**	GO:0010951	Negative Regulation Of Endopeptidase Activity	19	3.3	8.53E-06	4.43E-03
**PANTHER**	GO:0010951	Negative Regulation Of Endopeptidase Activity	33	2.0	5.99E-04	2.82E-02
**DAVID**	GO:0007399	Nervous System Development	77	1.8	9.50E-07	1.15E-03
**PANTHER**	GO:0007399	Nervous System Development	355	1.4	5.27E-10	3.41E-07
**DAVID**	GO:0001755	Neural Crest Cell Migration	15	3.1	2.11E-04	3.99E-02
**PANTHER**	GO:0001755	Neural Crest Cell Migration	18	2.9	3.27E-04	1.88E-02
**DAVID**	GO:0030335	Positive Regulation Of Cell Migration	51	1.9	1.76E-05	6.94E-03
**PANTHER**	GO:0030335	Positive Regulation Of Cell Migration	104	1.7	9.72E-07	1.74E-04
**DAVID**	GO:0032332	Positive Regulation Of Chondrocyte Differentiation	11	5.3	1.02E-05	4.43E-03
**PANTHER**	GO:0032332	Positive Regulation Of Chondrocyte Differentiation	11	4.7	1.90E-04	1.24E-02
**DAVID**	GO:0014911	Positive Regulation Of Smooth Muscle Cell Migration	12	5.2	3.64E-06	3.15E-03
**PANTHER**	GO:0014911	Positive Regulation Of Smooth Muscle Cell Migration	15	3.6	1.35E-04	9.48E-03
**DAVID**	GO:0007165	Signal Transduction	184	1.4	4.90E-06	3.71E-03
**PANTHER**	GO:0007165	Signal Transduction	652	1.2	6.07E-07	1.27E-04
**DAVID**	GO:0016126	Sterol Biosynthetic Process	9	4.8	2.30E-04	4.01E-02
**PANTHER**	GO:0016126	Sterol Biosynthetic Process	16	3.2	2.25E-04	1.41E-02
**DAVID**	GO:0042311	Vasodilation	14	3.5	7.29E-05	2.10E-02
**PANTHER**	GO:0042311	Vasodilation	17	3.2	1.87E-04	1.23E-02

Cellular Component

Database	ID	GO Term	Count	Fold Enrichment	P-Value	FDR
**DAVID**	GO:0030424	Axon	69	1.84	7.71E-07	1.20E-04
**PANTHER**	GO:0030424	Axon	110	1.5	1.13E-04	1.40E-02
**DAVID**	GO:0005737	Cytoplasm	660	1.12	2.91E-04	1.80E-02
**PANTHER**	GO:0005737	Cytoplasm	1482	1.08	4.21E-06	9.26E-04
**DAVID**	GO:0031012	Extracellular Matrix	71	2.65	2.45E-14	1.14E-11
**PANTHER**	GO:0031012	Extracellular Matrix	128	2.02	1.50E-11	2.98E-08
**DAVID**	GO:0005576	Extracellular Region	336	1.48	1.68E-14	1.14E-11
**PANTHER**	GO:0005576	Extracellular Region	604	1.25	1.20E-08	7.96E-06
**DAVID**	GO:0005615	Extracellular Space	284	1.39	3.41E-09	7.93E-07
**PANTHER**	GO:0005615	Extracellular Space	477	1.26	3.19E-07	1.05E-04
**DAVID**	GO:0043005	Neuron Projection	62	1.62	1.62E-04	1.08E-02
**PANTHER**	GO:0043005	Neuron Projection	208	1.34	6.99E-05	9.89E-03

Molecular Function

Database	Term	GO Term	Count	Fold Enrichment	P-Value	FDR
**DAVID**	GO:0005509	Calcium Ion Binding	126	1.54	7.46E-07	4.20E-04
**PANTHER**	GO:0005509	Calcium Ion Binding	128	1.56	5.89E-06	2.48E-03
**DAVID**	GO:0050840	Extracellular Matrix Binding	13	3.72	7.63E-05	1.61E-02
**PANTHER**	GO:0050840	Extracellular Matrix Binding	19	3.13	8.60E-05	2.29E-02
**DAVID**	GO:0005201	Extracellular Matrix Structural Constituent	35	2.32	3.72E-06	1.57E-03
**PANTHER**	GO:0005201	Extracellular Matrix Structural Constituent	39	2.09	1.00E-04	2.54E-02
**DAVID**	GO:0005539	Glycosaminoglycan Binding	12	4.23	4.10E-05	1.12E-02
**PANTHER**	GO:0005539	Glycosaminoglycan Binding	62	2.26	9.17E-08	1.16E-04
**DAVID**	GO:0008201	Heparin Binding	44	2.27	3.69E-07	4.20E-04
**PANTHER**	GO:0008201	Heparin Binding	47	2.36	1.10E-06	6.94E-04
**DAVID**	GO:0005515	Protein Binding	1477	1.07	7.38E-07	4.20E-04
**PANTHER**	GO:0005515	Protein Binding	1760	1.08	1.73E-09	2.91E-06

Supplemental Table S5 – DAVID and PANTHER GO Term Intersections of **Upregulated** Genes

Biological Process

Database	ID	GO Term	Count	Fold Enrichment	P-Value	FDR
**DAVID**	GO:0008608	Attachment Of Spindle Microtubules To Kinetochore	7	8.44	1.08E-04	2.24E-02
**PANTHER**	GO:0008608	Attachment Of Spindle Microtubules To Kinetochore	9	7.00	2.59E-05	3.83E-03
**DAVID**	GO:0051301	Cell Division	58	3.23	7.73E-15	2.56E-11
**PANTHER**	GO:0051301	Cell Division	76	2.95	2.73E-15	4.23E-12
**DAVID**	GO:0006695	Cholesterol Biosynthetic Process	11	6.28	5.93E-06	2.18E-03
**PANTHER**	GO:0006695	Cholesterol Biosynthetic Process	11	6.01	1.10E-05	1.78E-03
**DAVID**	GO:0007059	Chromosome Segregation	22	4.97	1.97E-09	2.17E-06
**PANTHER**	GO:0007059	Chromosome Segregation	65	3.96	1.11E-18	2.86E-15
**DAVID**	GO:0006260	Dna Replication	19	3.38	1.19E-05	3.38E-03
**PANTHER**	GO:0006260	Dna Replication	35	3.54	1.60E-09	8.27E-07
**DAVID**	GO:0006270	Dna Replication Initiation	10	7.75	2.79E-06	1.32E-03
**PANTHER**	GO:0006270	Dna Replication Initiation	11	7.94	1.20E-06	2.56E-04
**DAVID**	GO:0006268	Dna Unwinding Involved In Dna Replication	8	7.89	3.98E-05	9.42E-03
**PANTHER**	GO:0006268	Dna Unwinding Involved In Dna Replication	9	8.66	6.49E-06	1.12E-03
**DAVID**	GO:0000278	Mitotic Cell Cycle	26	3.64	4.31E-08	2.86E-05
**PANTHER**	GO:0000278	Mitotic Cell Cycle	95	3.12	2.50E-20	9.72E-17
**DAVID**	GO:0000281	Mitotic Cytokinesis	14	4.47	1.14E-05	3.38E-03
**PANTHER**	GO:0000281	Mitotic Cytokinesis	16	3.90	1.51E-05	2.32E-03
**DAVID**	GO:0007095	Mitotic G2 Dna Damage Checkpoint	10	5.56	5.43E-05	1.20E-02
**PANTHER**	GO:0007095	Mitotic G2 Dna Damage Checkpoint Signaling	10	5.77	3.67E-05	5.14E-03
**DAVID**	GO:0000070	Mitotic Sister Chromatid Segregation	14	7.99	7.10E-09	5.89E-06
**PANTHER**	GO:0000070	Mitotic Sister Chromatid Segregation	36	5.39	2.63E-14	3.41E-11
**DAVID**	GO:0007094	Mitotic Spindle Assembly Checkpoint	14	9.49	5.89E-10	9.77E-07
**PANTHER**	GO:0007094	Mitotic Spindle Assembly Checkpoint Signaling	13	8.76	5.16E-08	1.95E-05
**DAVID**	GO:0051256	Mitotic Spindle Midzone Assembly	7	13.81	3.55E-06	1.47E-03
**PANTHER**	GO:0051256	Mitotic Spindle Midzone Assembly	7	12.86	1.02E-05	1.71E-03
**DAVID**	GO:0007052	Mitotic Spindle Organization	14	5.24	1.76E-06	9.72E-04
**PANTHER**	GO:0007052	Mitotic Spindle Organization	22	4.68	2.50E-08	1.02E-05
**DAVID**	GO:0016126	Sterol Biosynthetic Process	8	9.14	1.33E-05	3.38E-03
**PANTHER**	GO:0016126	Sterol Biosynthetic Process	13	5.97	1.87E-06	3.92E-04

Cellular Component

Database	ID	GO Term	Count	Fold Enrichment	P-Value	FDR
**DAVID**	GO:0005814	Centriole	19	2.51	5.84E-04	1.89E-02
**PANTHER**	GO:0005814	Centriole	21	2.67	1.22E-04	7.54E-03

**DAVID**	GO:0005813	Centrosome	54	2.04	1.20E-06	1.25E-04
**PANTHER**	GO:0005813	Centrosome	63	1.96	1.43E-06	1.67E-04
**DAVID**	GO:0005694	Chromosome	27	2.26	1.68E-04	6.07E-03
**PANTHER**	GO:0005694	Chromosome	137	1.46	1.65E-05	1.49E-03
**DAVID**	GO:0000775	Chromosome, Centromeric Region	14	4.62	7.76E-06	5.29E-04
**PANTHER**	GO:0000775	Chromosome, Centromeric Region	41	3.41	1.71E-10	1.13E-07
**DAVID**	GO:0071162	Cmg Complex	6	11.89	7.34E-05	3.22E-03
**PANTHER**	GO:0071162	Cmg Complex	7	12.86	1.02E-05	1.01E-03
**DAVID**	GO:0000940	Condensed Chromosome Outer Kinetochore	8	13.41	5.60E-07	1.15E-04
**PANTHER**	GO:0000940	Condensed Chromosome Outer Kinetochore	9	12.99	4.99E-07	8.24E-05
**DAVID**	GO:0000779	Condensed Chromosome, Centromeric Region	6	8.17	5.73E-04	1.89E-02
**PANTHER**	GO:0000779	Condensed Chromosome, Centromeric Region	34	3.88	3.28E-10	1.62E-07
**DAVID**	GO:0045171	Intercellular Bridge	17	3.70	1.21E-05	7.42E-04
**PANTHER**	GO:0045171	Intercellular Bridge	16	3.44	5.73E-05	3.92E-03
**DAVID**	GO:0005871	Kinesin Complex	12	5.13	1.56E-05	8.73E-04
**PANTHER**	GO:0005871	Kinesin Complex	11	4.73	7.20E-05	4.75E-03
**DAVID**	GO:0000776	Kinetochore	29	3.90	1.25E-09	7.69E-07
**PANTHER**	GO:0000776	Kinetochore	30	3.65	1.22E-08	3.03E-06
**DAVID**	GO:0005874	Microtubule	32	2.09	1.56E-04	6.07E-03
**PANTHER**	GO:0005874	Microtubule	51	2.19	9.44E-07	1.17E-04
**DAVID**	GO:0015630	Microtubule Cytoskeleton	28	3.08	4.04E-07	1.15E-04
**PANTHER**	GO:0015630	Microtubule Cytoskeleton	123	1.80	1.42E-09	4.67E-07
**DAVID**	GO:0030496	Midbody	25	2.88	6.22E-06	4.77E-04
**PANTHER**	GO:0030496	Midbody	26	2.58	4.88E-05	3.58E-03
**DAVID**	GO:0072686	Mitotic Spindle	23	3.46	7.63E-07	1.17E-04
**PANTHER**	GO:0072686	Mitotic Spindle	32	3.57	6.41E-09	1.81E-06
**DAVID**	GO:0005819	Spindle	23	3.36	1.22E-06	1.25E-04
**PANTHER**	GO:0005819	Spindle	59	2.80	2.55E-11	2.53E-08
**DAVID**	GO:0005876	Spindle Microtubule	11	5.33	2.88E-05	1.36E-03
**PANTHER**	GO:0005876	Spindle Microtubule	19	4.68	2.20E-07	4.36E-05
**DAVID**	GO:0051233	Spindle Midzone	8	6.46	1.65E-04	6.07E-03
**PANTHER**	GO:0051233	Spindle Midzone	11	6.01	1.10E-05	1.04E-03
**DAVID**	GO:0000922	Spindle Pole	20	3.16	1.79E-05	9.15E-04
**PANTHER**	GO:0000922	Spindle Pole	23	2.70	4.98E-05	3.53E-03

Molecular Function

Database	ID	GO Term	Count	Fold Enrichment	P-Value	FDR
**DAVID**	GO:0005524	ATP Binding	120	1.63	1.02E-07	9.90E-05
**PANTHER**	GO:0005524	ATP Binding	126	1.71	2.19E-08	5.54E-05
**DAVID**	GO:0016887	ATP Hydrolysis Activity	46	2.17	1.67E-06	8.13E-04
**PANTHER**	GO:0016887	ATP Hydrolysis Activity	37	1.95	2.65E-04	4.79E-02

**DAVID**	GO:0003688	Dna Replication Origin Binding	7	9.13	6.25E-05	1.52E-02
**PANTHER**	GO:0003688	Dna Replication Origin Binding	7	8.84	6.40E-05	1.35E-02
**DAVID**	GO:0008017	Microtubule Binding	33	2.52	2.78E-06	9.03E-04
**PANTHER**	GO:0008017	Microtubule Binding	34	2.53	3.74E-06	1.45E-03

Supplemental Table S6 – DAVID and PANTHER GO Term Intersections of **Downregulated** Genes

Biological Process

Database	ID	GO Term	Count	Fold Enrichment	P-Value	FDR
**DAVID**	GO:0009060	Aerobic Respiration	15	3.82	2.73E-05	5.59E-03
**PANTHER**	GO:0009060	Aerobic Respiration	23	2.28	7.01E-04	2.15E-02
**DAVID**	GO:0048846	Axon Extension Involved In Axon Guidance	6	8.67	3.55E-04	3.34E-02
**PANTHER**	GO:0048846	Axon Extension Involved In Axon Guidance	6	8.66	3.00E-04	1.11E-02
**DAVID**	GO:0007411	Axon Guidance	34	3.02	2.32E-08	1.82E-05
**PANTHER**	GO:0007411	Axon Guidance	43	2.92	6.59E-09	1.26E-06
**DAVID**	GO:0031103	Axon Regeneration	7	7.14	2.58E-04	2.93E-02
**PANTHER**	GO:0031103	Axon Regeneration	9	5.72	1.19E-04	5.47E-03
**DAVID**	GO:0030509	Bmp Signaling Pathway	18	3.28	2.73E-05	5.59E-03
**PANTHER**	GO:0030509	Bmp Signaling Pathway	17	2.93	2.26E-04	9.13E-03
**DAVID**	GO:0001658	Branching Involved In Ureteric Bud Morphogenesis	12	4.73	2.93E-05	5.77E-03
**PANTHER**	GO:0001658	Branching Involved In Ureteric Bud Morphogenesis	14	4.83	8.13E-06	6.34E-04
**DAVID**	GO:0016339	Calcium-Dependent Cell-Cell Adhesion Via Plasma Membrane Cell Adhesion Molecules	18	6.50	5.90E-10	5.56E-07
**PANTHER**	GO:0016339	Calcium-Dependent Cell-Cell Adhesion Via Plasma Membrane Cell Adhesion Molecules	18	6.21	1.91E-08	3.23E-06
**DAVID**	GO:0060070	Canonical Wnt Signaling Pathway	20	3.40	5.10E-06	1.72E-03
**PANTHER**	GO:0060070	Canonical Wnt Signaling Pathway	19	2.85	1.41E-04	6.20E-03
**DAVID**	GO:0007155	Cell Adhesion	94	2.87	2.07E-20	9.75E-17
**PANTHER**	GO:0007155	Cell Adhesion	152	2.50	2.49E-22	1.29E-18
**DAVID**	GO:0016477	Cell Migration	32	1.99	3.39E-04	3.34E-02
**PANTHER**	GO:0016477	Cell Migration	110	1.96	2.73E-10	7.45E-08
**DAVID**	GO:0098609	Cell-Cell Adhesion	28	2.48	2.33E-05	5.43E-03
**PANTHER**	GO:0098609	Cell-Cell Adhesion	100	2.92	4.55E-19	1.18E-15
**DAVID**	GO:0007267	Cell-Cell Signaling	41	3.04	5.65E-10	5.56E-07
**PANTHER**	GO:0007267	Cell-Cell Signaling	136	1.98	9.96E-13	5.33E-10
**DAVID**	GO:0048706	Embryonic Skeletal System Development	9	4.87	3.61E-04	3.34E-02
**PANTHER**	GO:0048706	Embryonic Skeletal System Development	20	2.44	6.65E-04	2.06E-02
**DAVID**	GO:0007157	Heterophilic Cell-Cell Adhesion Via Plasma Membrane Cell Adhesion Molecules	15	4.73	1.94E-06	9.16E-04
**PANTHER**	GO:0007157	Heterophilic Cell-Cell Adhesion Via Plasma Membrane Cell Adhesion Molecules	15	4.41	9.85E-06	7.39E-04
**DAVID**	GO:0007156	Homophilic Cell Adhesion Via Plasma Membrane Adhesion Molecules	48	4.78	1.32E-19	3.12E-16
**PANTHER**	GO:0007156	Homophilic Cell Adhesion Via Plasma Membrane Adhesion Molecules	47	4.44	4.08E-15	3.73E-12
**DAVID**	GO:0006954	Inflammatory Response	49	1.95	1.30E-05	3.60E-03
**PANTHER**	GO:0006954	Inflammatory Response	62	1.76	6.38E-05	3.26E-03

**DAVID**	GO:0030324	Lung Development	15	3.13	2.61E-04	2.93E-02
**PANTHER**	GO:0030324	Lung Development	28	2.43	8.84E-05	4.28E-03
**DAVID**	GO:0030225	Macrophage Differentiation	9	4.87	3.61E-04	3.34E-02
**PANTHER**	GO:0030225	Macrophage Differentiation	12	4.54	5.99E-05	3.14E-03
**DAVID**	GO:0042776	Mitochondrial Atp Synthesis Coupled Proton Transport	16	4.27	3.20E-06	1.26E-03
**PANTHER**	GO:0042776	Mitochondrial Atp Synthesis Coupled Proton Transport	16	3.97	1.55E-05	1.08E-03
**DAVID**	GO:0006120	Mitochondrial Electron Transport, Nadh To Ubiquinone	11	4.14	2.37E-04	2.93E-02
**PANTHER**	GO:0006120	Mitochondrial Electron Transport, Nadh To Ubiquinone	11	3.80	4.49E-04	1.52E-02
**DAVID**	GO:0032981	Mitochondrial Respiratory Chain Complex I Assembly	13	3.47	2.99E-04	3.24E-02
**PANTHER**	GO:0032981	Mitochondrial Respiratory Chain Complex I Assembly	13	3.33	4.25E-04	1.45E-02
**DAVID**	GO:0043066	Negative Regulation Of Apoptotic Process	53	1.69	2.21E-04	2.90E-02
**PANTHER**	GO:0043066	Negative Regulation Of Apoptotic Process	95	1.70	2.24E-06	2.01E-04
**DAVID**	GO:0090090	Negative Regulation Of Canonical Wnt Signaling Pathway	26	2.64	1.66E-05	4.12E-03
**PANTHER**	GO:0090090	Negative Regulation Of Canonical Wnt Signaling Pathway	24	2.76	3.06E-05	1.89E-03
**DAVID**	GO:0008285	Negative Regulation Of Cell Proliferation	49	1.84	5.80E-05	1.05E-02
**PANTHER**	GO:0008285	Negative Regulation Of Cell Proliferation	82	1.84	7.07E-07	7.79E-05
**DAVID**	GO:0021675	Nerve Development	8	6.60	1.18E-04	1.80E-02
**PANTHER**	GO:0021675	Nerve Development	18	3.07	8.95E-05	4.30E-03
**DAVID**	GO:0007399	Nervous System Development	60	2.45	3.14E-10	4.93E-07
**PANTHER**	GO:0007399	Nervous System Development	251	1.79	3.03E-18	5.89E-15
**DAVID**	GO:0001649	Osteoblast Differentiation	19	2.57	3.96E-04	3.59E-02
**PANTHER**	GO:0001649	Osteoblast Differentiation	22	2.48	3.03E-04	1.11E-02
**DAVID**	GO:0090179	Planar Cell Polarity Pathway Involved In Neural Tube Closure	6	9.45	2.18E-04	2.90E-02
**PANTHER**	GO:0090179	Planar Cell Polarity Pathway Involved In Neural Tube Closure	6	7.94	4.28E-04	1.45E-02
**DAVID**	GO:0048711	Positive Regulation Of Astrocyte Differentiation	6	8.67	3.55E-04	3.34E-02
**PANTHER**	GO:0048711	Positive Regulation Of Astrocyte Differentiation	6	7.33	5.94E-04	1.88E-02
**DAVID**	GO:0030513	Positive Regulation Of Bmp Signaling Pathway	11	4.14	2.37E-04	2.93E-02
**PANTHER**	GO:0030513	Positive Regulation Of Bmp Signaling Pathway	9	3.76	1.55E-03	3.88E-02
**DAVID**	GO:0030335	Positive Regulation Of Cell Migration	39	2.53	2.38E-07	1.40E-04
**PANTHER**	GO:0030335	Positive Regulation Of Cell Migration	79	2.35	8.59E-11	2.84E-08
**DAVID**	GO:0008284	Positive Regulation Of Cell Proliferation	63	2.00	2.37E-07	1.40E-04
**PANTHER**	GO:0008284	Positive Regulation Of Cell Proliferation	106	1.78	6.73E-08	9.42E-06
**DAVID**	GO:0032332	Positive Regulation Of Chondrocyte Differentiation	8	6.60	1.18E-04	1.80E-02
**PANTHER**	GO:0032332	Positive Regulation Of Chondrocyte Differentiation	8	6.05	2.08E-04	8.58E-03
**DAVID**	GO:0070374	Positive Regulation Of Erk1 And Erk2 Cascade	28	2.11	3.57E-04	3.34E-02

**PANTHER**	GO:0070374	Positive Regulation Of Erk1 And Erk2 Cascade	28	2.07	8.37E-04	2.46E-02
**DAVID**	GO:0048146	Positive Regulation Of Fibroblast Proliferation	12	3.71	3.02E-04	3.24E-02
**PANTHER**	GO:0048146	Positive Regulation Of Fibroblast Proliferation	11	3.23	1.41E-03	3.64E-02
**DAVID**	GO:0010628	Positive Regulation Of Gene Expression	60	2.00	4.76E-07	2.50E-04
**PANTHER**	GO:0010628	Positive Regulation Of Gene Expression	113	1.53	2.40E-05	1.55E-03
**DAVID**	GO:0045669	Positive Regulation Of Osteoblast Differentiation	16	3.65	2.42E-05	5.43E-03
**PANTHER**	GO:0045669	Positive Regulation Of Osteoblast Differentiation	18	4.20	2.34E-06	2.08E-04
**DAVID**	GO:0050731	Positive Regulation Of Peptidyl-Tyrosine Phosphorylation	17	3.07	1.13E-04	1.80E-02
**PANTHER**	GO:0050731	Positive Regulation Of Peptidyl-Tyrosine Phosphorylation	25	2.19	8.53E-04	2.50E-02
**DAVID**	GO:0001934	Positive Regulation Of Protein Phosphorylation	27	2.07	6.20E-04	4.96E-02
**PANTHER**	GO:0001934	Positive Regulation Of Protein Phosphorylation	76	1.70	2.86E-05	1.79E-03
**DAVID**	GO:0014911	Positive Regulation Of Smooth Muscle Cell Migration	10	7.53	2.67E-06	1.14E-03
**PANTHER**	GO:0014911	Positive Regulation Of Smooth Muscle Cell Migration	12	5.15	2.12E-05	1.43E-03
**DAVID**	GO:0048661	Positive Regulation Of Smooth Muscle Cell Proliferation	13	3.82	1.14E-04	1.80E-02
**PANTHER**	GO:0048661	Positive Regulation Of Smooth Muscle Cell Proliferation	17	3.18	9.81E-05	4.65E-03
**DAVID**	GO:0086091	Regulation Of Heart Rate By Cardiac Conduction	10	4.56	2.43E-04	2.93E-02
**PANTHER**	GO:0086091	Regulation Of Heart Rate By Cardiac Conduction	10	3.87	7.09E-04	2.17E-02
**DAVID**	GO:0051384	Response To Glucocorticoid	12	3.47	5.63E-04	4.74E-02
**PANTHER**	GO:0051384	Response To Glucocorticoid	26	2.89	7.30E-06	5.78E-04
**DAVID**	GO:0042542	Response To Hydrogen Peroxide	11	4.24	1.95E-04	2.71E-02
**PANTHER**	GO:0042542	Response To Hydrogen Peroxide	21	3.33	8.21E-06	6.31E-04
**DAVID**	GO:0055093	Response To Hyperoxia	7	7.14	2.58E-04	2.93E-02
**PANTHER**	GO:0055093	Response To Hyperoxia	8	6.05	2.08E-04	8.55E-03
**DAVID**	GO:0001666	Response To Hypoxia	23	2.25	5.60E-04	4.74E-02
**PANTHER**	GO:0001666	Response To Hypoxia	36	2.10	1.24E-04	5.60E-03
**DAVID**	GO:0002931	Response To Ischemia	13	3.41	3.47E-04	3.34E-02
**PANTHER**	GO:0002931	Response To Ischemia	13	3.62	2.10E-04	8.57E-03
**DAVID**	GO:0032496	Response To Lipopolysaccharide	22	2.56	1.31E-04	1.93E-02
**PANTHER**	GO:0032496	Response To Lipopolysaccharide	42	2.06	4.41E-05	2.51E-03
**DAVID**	GO:0014070	Response To Organic Cyclic Compound	14	3.68	8.50E-05	1.49E-02
**PANTHER**	GO:0014070	Response To Organic Cyclic Compound	96	1.75	6.15E-07	6.97E-05
**DAVID**	GO:0007165	Signal Transduction	114	1.51	9.36E-06	2.94E-03
**PANTHER**	GO:0007165	Signal Transduction	391	1.29	4.67E-08	6.78E-06
**DAVID**	GO:0007416	Synapse Assembly	16	3.80	1.46E-05	3.81E-03
**PANTHER**	GO:0007416	Synapse Assembly	23	3.23	4.87E-06	4.05E-04
**DAVID**	GO:0042311	Vasodilation	12	4.52	4.59E-05	8.66E-03

**PANTHER**	GO:0042311	Vasodilation	13	4.30	4.76E-05	2.64E-03
**DAVID**	GO:0042060	Wound Healing	17	3.01	1.45E-04	2.07E-02
**PANTHER**	GO:0042060	Wound Healing	44	2.07	2.47E-05	1.59E-03

Cellular Component

Database	ID	GO Term	Count	Fold Enrichment	P-Value	FDR
**DAVID**	GO:0030424	Axon	49	2.28	1.60E-07	1.34E-05
**PANTHER**	GO:0030424	Axon	76	1.85	1.33E-06	2.39E-04
**DAVID**	GO:0009986	Cell Surface	70	1.84	1.23E-06	8.20E-05
**PANTHER**	GO:0009986	Cell Surface	94	1.63	1.54E-05	2.04E-03
**DAVID**	GO:0030425	Dendrite	55	2.07	6.06E-07	4.49E-05
**PANTHER**	GO:0030425	Dendrite	67	1.69	1.13E-04	5.31E-03
**DAVID**	GO:0005788	Endoplasmic Reticulum Lumen	35	1.94	2.81E-04	9.86E-03
**PANTHER**	GO:0005788	Endoplasmic Reticulum Lumen	37	1.86	8.32E-04	2.84E-02
**DAVID**	GO:0070062	Extracellular Exosome	162	1.25	1.91E-03	4.71E-02
**PANTHER**	GO:0070062	Extracellular Exosome	169	1.28	1.63E-03	4.96E-02
**DAVID**	GO:0031012	Extracellular Matrix	48	3.20	2.28E-12	3.80E-10
**PANTHER**	GO:0031012	Extracellular Matrix	82	2.31	5.33E-11	3.52E-08
**DAVID**	GO:0005576	Extracellular Region	218	1.70	1.31E-15	8.74E-13
**PANTHER**	GO:0005576	Extracellular Region	380	1.41	2.13E-12	4.21E-09
**DAVID**	GO:0005615	Extracellular Space	196	1.70	5.21E-14	1.16E-11
**PANTHER**	GO:0005615	Extracellular Space	308	1.46	2.06E-11	2.04E-08
**DAVID**	GO:0098982	Gaba-Ergic Synapse	16	3.18	1.29E-04	4.82E-03
**PANTHER**	GO:0098982	GABA-Ergic Synapse	16	2.85	4.46E-04	1.64E-02
**DAVID**	GO:0098978	Glutamatergic Synapse	54	2.19	1.22E-07	1.16E-05
**PANTHER**	GO:0098978	Glutamatergic Synapse	52	1.92	3.40E-05	3.07E-03
**DAVID**	GO:0043202	Lysosomal Lumen	15	2.64	1.49E-03	3.98E-02
**PANTHER**	GO:0043202	Lysosomal Lumen	17	2.75	4.32E-04	1.61E-02
**DAVID**	GO:0016020	Membrane	259	1.21	4.41E-04	1.40E-02
**PANTHER**	GO:0016020	Membrane	714	1.16	2.42E-07	5.98E-05
**DAVID**	GO:0045121	Membrane Raft	33	2.29	1.95E-05	9.89E-04
**PANTHER**	GO:0045121	Membrane Raft	43	2.06	3.43E-05	2.95E-03
**DAVID**	GO:0005743	Mitochondrial Inner Membrane	51	1.78	8.75E-05	3.65E-03
**PANTHER**	GO:0005743	Mitochondrial Inner Membrane	52	1.66	9.01E-04	3.02E-02
**DAVID**	GO:0005747	Mitochondrial Respiratory Chain Complex I	13	4.49	2.08E-05	9.89E-04
**PANTHER**	GO:0005747	Mitochondrial Respiratory Chain Complex I	13	4.21	5.70E-05	3.42E-03
**DAVID**	GO:0043005	Neuron Projection	46	2.12	2.89E-06	1.61E-04
**PANTHER**	GO:0043005	Neuron Projection	136	1.57	8.12E-07	1.61E-04
**DAVID**	GO:0043204	Perikaryon	24	2.54	6.63E-05	2.95E-03
**PANTHER**	GO:0043204	Perikaryon	24	2.43	1.97E-04	8.12E-03
**DAVID**	GO:0005886	Plasma Membrane	396	1.25	8.91E-08	9.91E-06

**PANTHER**	GO:0005886	Plasma Membrane	454	1.23	8.03E-07	1.77E-04
**DAVID**	GO:0014069	Postsynaptic Density	37	2.37	2.58E-06	1.57E-04
**PANTHER**	GO:0014069	Postsynaptic Density	45	2.07	1.86E-05	2.04E-03
**DAVID**	GO:0045211	Postsynaptic Membrane	27	2.29	1.30E-04	4.82E-03
**PANTHER**	GO:0045211	Postsynaptic Membrane	39	2.19	2.24E-05	2.22E-03
**DAVID**	GO:0070469	Respirasome	8	4.61	1.34E-03	3.72E-02
**PANTHER**	GO:0070469	Respirasome	19	3.05	6.37E-05	3.60E-03

Molecular Function

Database	ID	GO Term	Count	Fold Enrichment	P-Value	FDR
**DAVID**	GO:0005509	Calcium Ion Binding	84	1.82	1.34E-07	5.69E-05
**PANTHER**	GO:0005509	Calcium Ion Binding	85	1.86	2.76E-07	1.16E-04
**DAVID**	GO:1904929	Coreceptor Activity Involved In Wnt Signaling Pathway, Planar Cell Polarity Pathway	5	13.63	1.88E-04	2.99E-02
**PANTHER**	GO:1904929	Coreceptor Activity Involved In Wnt Signaling Pathway, Planar Cell Polarity Pathway	5	13.23	2.48E-04	4.32E-02
**DAVID**	GO:0005125	Cytokine Activity	34	2.90	6.16E-08	3.92E-05
**PANTHER**	GO:0005125	Cytokine Activity	44	2.99	2.39E-09	4.03E-06
**DAVID**	GO:0008083	Growth Factor Activity	32	3.13	2.47E-08	3.13E-05
**PANTHER**	GO:0008083	Growth Factor Activity	32	3.12	1.51E-07	6.96E-05
**DAVID**	GO:0008201	Heparin Binding	31	2.83	4.26E-07	1.35E-04
**PANTHER**	GO:0008201	Heparin Binding	34	3.05	9.85E-08	4.98E-05
**DAVID**	GO:0008137	Nadh Dehydrogenase (Ubiquinone) Activity	13	4.73	1.11E-05	2.36E-03
**PANTHER**	GO:0008137	Nadh Dehydrogenase (Ubiquinone) Activity	11	4.26	1.93E-04	3.49E-02
**DAVID**	GO:0005515	Protein Binding	844	1.09	2.88E-06	7.32E-04
**PANTHER**	GO:0005515	Protein Binding	988	1.09	1.00E-06	3.90E-04
**DAVID**	GO:0005102	Receptor Binding	45	1.79	2.30E-04	3.24E-02
**PANTHER**	GO:0005102	Signaling Receptor Binding	156	1.63	9.08E-09	7.66E-06
**DAVID**	GO:0017147	Wnt-Protein Binding	10	5.11	8.94E-05	1.62E-02
**PANTHER**	GO:0017147	Wnt-Protein Binding	10	4.96	1.33E-04	2.68E-02
